# A Pilot Observational Study Assessing Long-Term Changes in Clinical Parameters, Functional Capacity and Fall Risk of Patients With Chronic Renal Disease Scheduled for Hemodialysis

**DOI:** 10.3389/fmed.2022.682198

**Published:** 2022-02-04

**Authors:** Damiano D. Zemp, Olivier Giannini, Pierluigi Quadri, Mauro Tettamanti, Lorenzo Berwert, Soraya Lavorato, Silvio Pianca, Curzio Solcà, Eling D. de Bruin

**Affiliations:** ^1^Department of Health Sciences and Technology, Institute of Human Movement Sciences and Sport, ETH Zurich, Zurich, Switzerland; ^2^Service of Geriatrics, EOC, Ospedale Regionale di Mendrisio EOC, Mendrisio, Switzerland; ^3^Department of Medicine, EOC, Bellinzona, Switzerland; ^4^Division of Nephrology, EOC, Lugano, Switzerland; ^5^Faculty of Biomedical Sciences, Università della Svizzera italiana, Lugano, Switzerland; ^6^Department of Neuroscience, Istituto di Ricerche Farmacologiche Mario Negri IRCCS, Milano, Italy; ^7^Service of Nephrology, Centro Dialisi Nefrocure e Clinica Luganese Moncucco, Lugano, Switzerland; ^8^Department of Neurobiology, Care Sciences and Society, Karolinska Institute, Stockholm, Sweden; ^9^Department of Health, OST - Eastern Swiss University of Applied Sciences, St. Gallen, Switzerland

**Keywords:** chronic kidney disease, end stage renal disease, hemodialysis (HD), physical activity, motor capacity, cognitive capacity, falls, frailty

## Abstract

**Background:**

Patients with end-stage renal disease are known to be particularly frail, and the cause is still widely seen as being directly related to specific factors in renal replacement therapy. However, a closer examination of the transitional phase from predialysis to long-term hemodialysis leads to controversial explanations, considering that the frailty process is already well-described in the early stages of renal insufficiency. This study aims to describe longitudinally and multifactorially changes in the period extending from the decision to start the replacement therapy through to the end of 2 years of hemodialysis. We hypothesized that frailty is pre-existent in the predialysis phase and does not worsen with the beginning of the replacement therapy. Between 2015 and 2018 we recruited 25 patients (72.3 ± 5.7 years old) in a predialysis program, with the expectation that replacement therapy would begin within the coming few months.

**Methods:**

The patients underwent a baseline visit before starting hemodialysis, with 4 follow-up visits in the first 2 years of treatment. Health status, physical performance, cognitive functioning, hematology parameters, and adverse events were monitored during the study period.

**Results:**

At baseline, our sample had a high variability with patients ranging from extremely frail to very fit. In the 14 participants that did not drop out of the study, out of 32 clinical and functional measures, a statistically significant worsening was only observed in the Short Physical Performance Battery (SPPB) score (*p* < 0.01, *F* = 8.50) and the number of comorbidities (*p* = 0.01, *F* = 3.94). A careful analysis, however, reveals a quite stable situation in the first year of replacement therapy, for both frail and fit participants and a deterioration in the second year that in frail participants could lead to death.

**Conclusion:**

Our results should stimulate a reassessment about the role of a predialysis program in reducing complications during the transitional phase, but also about frailty prevention programs once hemodialysis has begun, for both frail and fit patients, to maintain satisfactory health status.

## Introduction

Worldwide, about 300 persons out of every million suffer from end-stage renal disease (ESRD) and receive dialysis (1) which implies that 0.1% of the world population is living with ESRD (2). For 84% of patients in Europe, the first choice of renal replacement therapy (RRT) is hemodialysis (HD) (3), which is also the most used treatment worldwide. In 2015, Switzerland registered about 4,500 patients on HD with an average age of 68 years, and 50% being older than 71 years (4). The median life expectancy in Switzerland for patients beginning RRT is about 6 years (4). Compared with the age-matched general population in Switzerland with an average survival expectancy of about 20 years (5), the population on RRT is particularly frail (6–8). With the increase in the age of patients with HD due to better accessibility to and better quality of treatment, frailty in the ESRD population is expected to become a major topic to be dealt with in clinical practice (9).

Many studies have explained the higher rate of frailty through HD specific factors, such as a less active lifestyle (10–12) or reduced physical performance (13, 14), although many other factors related to chronic kidney disease (CKD) may also influence frailty; e.g., comorbid conditions, uremic neuropathy, osteopathy, weakness (15), inflammatory status (16), reduced cardiovascular function (17, 18), or cognitive function (19).

As falls are important indicators related to higher frailty levels, it is not surprising that fall rates are higher in the HD population, similar to frail elderly (20–24) when compared to the general population.

Recent studies suggest that patients with CKD are frailer (25) and have more fall risk factors, such as gait disorders (26), balance impairment (27, 28), or cognitive impairment (29), already in the early stages of the renal disease; however, it is as of yet not clear whether HD itself represents a factor for higher fall risk. Although two recent studies have attempted to shed a light on the development of patients into frailty status in the first years of HD, analyzing fall rate (30) and physical performance (16), HD did not result to be the main cause of decreasing health status.

Although frailty and falls are an issue also in young adults on HD (21, 22, 31), our study focuses on the elderly population to try to gain a better understanding of the role of the transitional phase from predialysis to long-term dialysis in the frailty development process in patients with CKD, a field of study that is not yet well understood and remains controversial, as has been reported in a recent review (32).

The objective of this study is to longitudinally describe the evolution of fall-related factors, health status, and functional capacity of patients expected to receive HD. Patients with ESRD were followed from the 6-month predialysis phase through to 2 years after starting HD. We are convinced that a better understanding of the processes that lead to higher frailty in patients with CKD could be useful for developing specific preventive strategies for this population. The period of 2 years was chosen to have enough time to reveal possible changes due to the RRT and because similar studies limited their analysis to the acute impact of HD on falls (30) and frailty (16) in the first year of therapy.

## Materials and Methods

### Study Design

This longitudinal pilot observational study is a multicentric project in an ambulant setting of patients with CKD having ESRD.

The study protocol included functional and neuropsychological tests, health-related questionnaires, and monitoring of health status through monthly calls and blood analysis. Baseline assessment was made when the commencement of HD within 6 months was predicted by the treating medical doctor. At this moment the patient was informed and in most cases, the arteriovenous fistula was created to be ready once dialysis is needed. After the first HD session, the follow-up phase began and included 4 visits: at 3, 6, 12, and 24 months after the first HD. The patients who did not start HD as expected after the baseline assessment were re-tested at one-year intervals until HD started. At least 24 h after the end of an HD session, assessments took place at the dialysis center where the participant received treatment.

### Participants and Settings

Patients were recruited in Canton Ticino—Switzerland, from three HD units of the nephrology department of the multicentric public hospital (Ente Ospedaliero Cantonale, in the towns of Mendrisio, Lugano, and Bellinzona), and the private dialysis center Nefrocure in Lugano, between 2015 and 2018. The baseline assessment of the first participant was in January 2015, and the final follow-up assessment of the last participant was in June 2020. Inclusion criteria were CKD 5 [estimated glomerular filtration rate (eGFR) <20 ml/min/1.73 m^2^] with eligibility criteria for an HD program, ability to understand information for executing assessments, and ability to walk autonomously. Exclusion criteria were unstable or preterminal health status (e.g., recent surgery and ongoing oncological treatment), diagnosis of dementia [Clinical Dementia Rating Scale ≥ 1 (33)], and diagnosis of depressive syndromes.

Nephrologists that had patients with CKD in their care asked the ones with ESRD, who were expected to enter dialysis in the upcoming 6 months, about their interest in participating in the longitudinal study. The names and phone numbers of patients who consented to partake were sent to the principal investigator. They were then contacted by the researcher for a baseline visit that included the control of inclusion and exclusion criteria, the written and oral explanation of the study, and the signing of the written informed consent form before starting with the assessments.

### Variables

At baseline, general characteristics were recorded: age, gender, body mass index (34), education, and household situation.

Functional tests consisted of three widely used and reliable assessment batteries: the Expanded Timed Get up and Go Test (ETGUG) (35), the Performance Oriented Mobility Assessment (POMA) (36, 37), and the Short Physical Performance Battery (SPPB) (38, 39).

Muscle strength was tested using a Jamar® hydraulic hand dynamometer (Performance Health International LTD, Suton-in-Ashfield, UK) for handgrip (40, 41) and Nicholas MMT (Model 01160, Lafayette Instrument, Lafayette, USA), a manual muscle tester, for hip flexion (42, 43).

Gait speed was determined by instrumental gait analysis assessed on a 10 m pathway *via* a triaxial accelerometer affixed to the lower trunk (DynaPort MiniMod, McRoberts, The Hague, Netherlands) (44–46). To register gait speed as a marker of mobility (47, 48), and neurological system-related discrete gait parameters (49) the protocol requires a steady-state walking speed. Therefore, the participants started at least 2 m before and stopped at least 2 m after the 10 m pathway so that the acceleration and deceleration phases were not included in the analysis. In this article, we focus on mobility and clinical aspects. A complete analysis of neurological system-related discrete gait parameters was recently published (50). Both ETGUG and self-selected gait speed were assessed under single- and dual-task conditions, to describe attentional demand (51, 52). The cognitive task to be performed under dual-task conditions consisted of counting down from 100 in steps of 3.

Several questionnaires were used to assess the health status of the participants. The Short-Form Health Survey (SF-12) assesses mental and physical health aspects of quality of life (53–55). For functional evaluation, the Barthel Index of activities of daily living (ADL) (56, 57) and the Nottingham Extended Activities of Daily Living Scale (EADS) (58) were used. In addition, the pain was assessed with the Visual Analog Scale for Pain (VAS-P) (59, 60), depression with the 10-item Geriatric Depression Scale (GDS-10) (61, 62), and fatigue with the Multidimensional Fatigue Inventory (MFI-20) (63, 64). At each visit, participants received a pedometer (Step Watch^TM^, Modus, Washington DC, USA) for seven consecutive days that objectively measured their physical activity level (65–67). The device was attached to the right ankle and measured the numbers of right steps during the defined period. The number of right steps was doubled to have an output to be compared with international normative data (68).

Cognitive status was assessed by a neuropsychologist using the Mini-Mental State Examination for global cognition (69, 70), the Frontal Assessment Battery (FAB) for executive functions (71, 72), and the Trail Making Test A and B (TMT-A, TMT-B) for neurological and neuropsychological status (73, 74). For FAB and TMT, the equivalent score was calculated adjusting the test score for age and schooling (75).

The Cumulative Illness Rating Scale was used to monitor the number (CIRS-C) and the severity (CIRS-S) of comorbidities (76–78) throughout the years. The health status was monitored with blood samples taken monthly at the beginning of the dialytic session after the long interdialytic pause, and a monthly call to record falls [according to the WHO (79)] and adverse events [according to the Human Research Ordinance (HRO) (80)]. At baseline, participants were asked about adverse events and falls in the previous 12 months. Supplementary Material S1 lists all assessments with the reference value and description.

Based on tests quantifying indicators such as gait speed, and handgrip strength, an estimate for the fall risk and the probability of sarcopenia could be made.

For sarcopenia, we used the indicators defined by the European Working Group on Sarcopenia published in 2018 (40): handgrip < 16 kg for women and < 27 kg for men, time for 5 times chair rise > 15 s or unable, SPPB score ≤ 8 points, gait speed < 0.8 m/s. For fall risk we used widely used and accepted indicators published in the last decade (35, 36, 81–83): ETGUG total time > 34 s, SPPB score < 8 points, POMA score < 20 points, gait speed < 0.80 m/s, and at least one fall in the previous 12 months.

### Statistical Methods

Due to the explorative character of the study and the lack of previous similar research, no power analysis was made. The reference population for this study is about 350,000 inhabitants, and in the dialysis centers yearly about 50 new dialysis patients are enrolled. Excluding patients with other RRT as HD, those not involved in a predialysis program, and those not fulfilling the inclusion/exclusion criteria, we estimated being able to recruit 7–10 patients per year for a total of 30–40 participants.

Raw data were inserted anonymously into a database from where they were extracted for statistical analysis. Descriptive statistics were calculated for participant characteristics, health status, functional capacity, cognitive function, and hematology parameters at baseline and at each follow-up session. The dual-task cost for gait speed was calculated in percent using the formula:


$$\begin{array}{l} \left[100-100\;x\;dual-task\;value/single\;task\;value\right]. \end{array}$$

Baseline characteristics were reported both for completers (*n* = 14) and dropouts (*n* = 11). Two subgroups beneath the dropouts (participants that died and participants that did not start HD because of the stabilization of their renal function) were reported in the additional file Supplementary Material S2.

In the longitudinal analysis, for each parameter, mean deviation and SD were calculated and plotted on a line graph, also containing data of each participant, to make evident the progression of the parameter. Regression analysis for each parameter was made by one-way repeated-measures ANOVA. Where sphericity was not met, nonparametric tests were used. A step-by-step analysis was made with the tests for within-subjects contrasts to analyze the changes between each interval.

To determine whether some baseline characteristics can influence the evolution of health status, participants with more than one factor indicating a risk of falls or sarcopenia, a fall reported in the last year, or subnormal parameters of some baseline measure (ETGUG total time, SPPB total score, handgrip strength, gait speed, and albumin and CRP level) were defined as frail. Participants with normal values, no falls reported and not more than one factor of fall risk or sarcopenia were defined as fit.

All participants who were not examined at the two-year follow-up were excluded from the longitudinal analysis, as the aim of this study was to analyze patients on long-term dialysis and not to analyze the acute effects of RRT on patients new to HD. Therefore, drop-outs were analyzed in a descriptive form. Missing data, e.g., due to technical problems or health status of the participants, was not replaced and no adjustment of mean and standard deviation was carried out. The significance cut-off was set at *p* = 0.05.

For statistical analysis, IBM SPSS Statistics 26 was used.

## Results

### Participants

In the analyzed period (January 2015–June 2018), 151 patients started dialysis, 23 were excluded because they started peritoneal dialysis. Of the 127 remainings, 23 were a late referral and were not in a predialysis program (early referral). Of the 104 candidates, only 27 fulfilled inclusion/exclusion criteria and were accepted to participate in the study. Unfortunately, no data are available about the 77 patients that were not referred to the principal investigator, in particular, if they were excluded or if they fulfilled exclusion/inclusion criteria but denied to participate. This lack of information does not permit us to know if the health status of our sample is representative of the predialysis population of our region.

**Figure 1:**
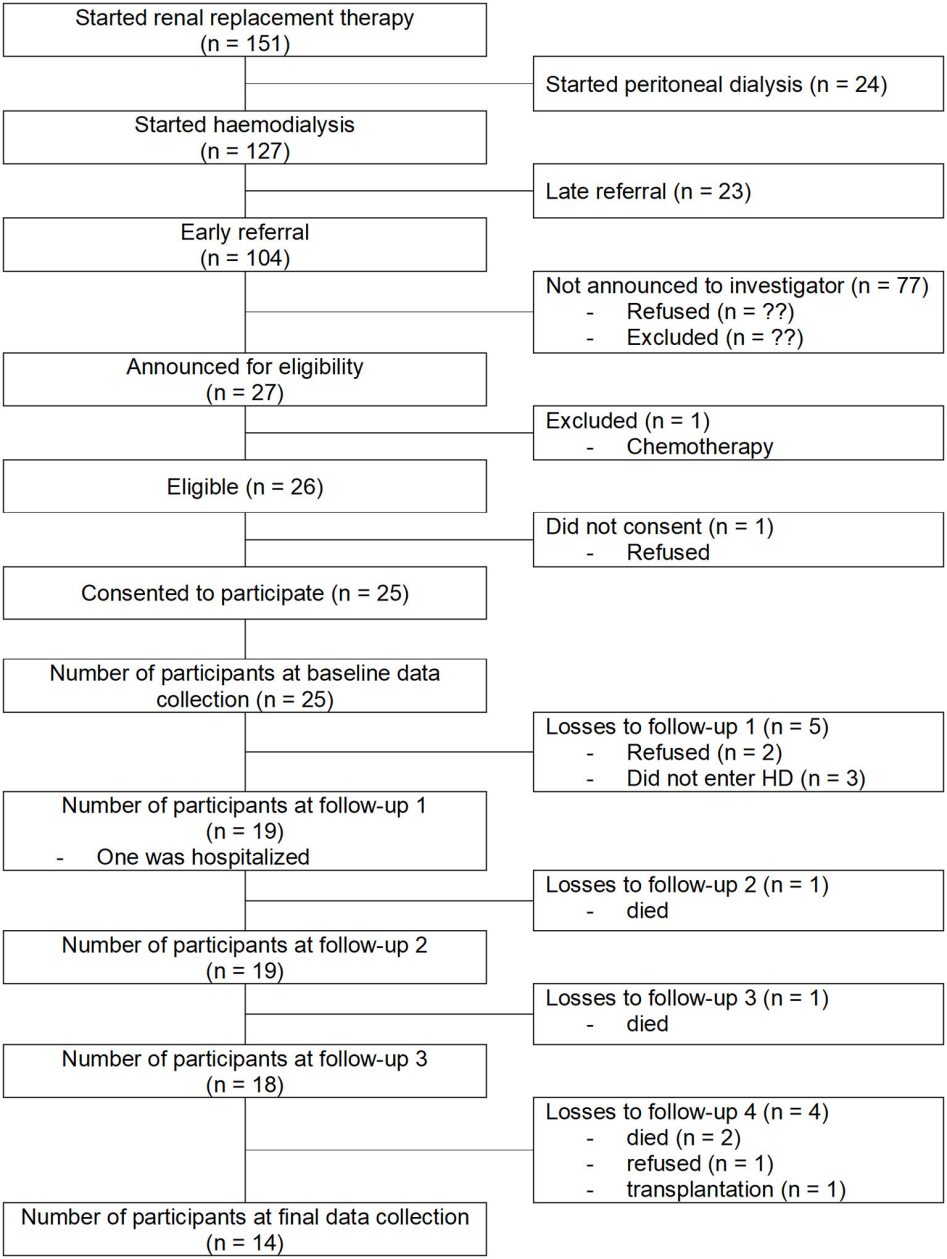
Study flow diagram.

A total of 27 patients were recruited by the nephrologist. Two patients were excluded at the first visit. The first was excluded because of cancer that was diagnosed after the recruitment and needed chemotherapy, and the second decided not to participate after having received the oral and written information. Out of the 25 that signed the written consent, 11 dropped out. Two individuals refused to continue after the baseline assessment, four died (two in the first year of HD and 2 in the second year of HD). In three patients that were expected to enter HD in the following months, the renal function stabilized and HD was no longer needed until the end of this study. One individual received a kidney transplant in the 2nd year of HD, one declined to continue after the first year of follow up. In the end, 14 participants concluded the study with at least one visit before entering HD and 4 visits at 3, 6, 12, and 24 months after the first HD. Figure 1 shows the study flow diagram.

Due to the high heterogeneity of the population, we found some people were cognitively and physically very frail (low gait speed, low SPPB score, reported several falls, and low FAB score), others were very fit and had excellent cognitive functions, while the remainder lay somewhere in between.

Table 1 shows the demographic, clinical, and functional data of the recruited patients by groups: concluders vs. dropouts.

**Table 1:** Main demographic, clinical and functional data [mean ± SD (min–max)] at baseline by groups.

	**Completers**	**Dropouts**	**Reference**
	**(*n* = 14)**	**(*n* = 11)**	**value**
**General characteristics**			
Gender (M/W)	7 / 7	5 / 6	
Age (years)	72.4 ± 5.4 [60–81]	76.6 ± 6.5 [64–86]	
BMI (kg/m^2^)	29.7 ± 3.6 [22.6–35.1]	28.9 ± 6.5 [16.7–40.2]	18.5–24.9
Schooling (years)	8.2 ± 3.3 [5–17]	8.2 ± 3.8 [3–17]	
Household (alone / with others)	6 / 8	4 / 7	
**Health status**			
Physical health	36.5 ± 5.9 [25.0–49.6]	40.3 ± 10.0 [23.4–54.2]	> 40
Mental health	49.3 ± 9.6 [28.3–65.1]	54.7 ± 8.1 [34.1–63.6]	> 40
Autonomy	96.4 ± 5.0 [85–100]	97.3 ± 5.2 [85–100]	≥ 75
Independence	15.6 ± 3.4 [9–20]	17.0 ± 3.3 [12–22]	≥ 17
General fatigue	12.3 ± 3.4 [8–19]	10.8 ± 4.4 [5–17]	> 10
Pain	42.5 ± 24.9 [5–100]	26.8 ± 29.9 [0–100]	<40
GDS-10	2.9 ± 2.2 [0–7]	3.4 ± 3.3 [0–10]	≤ 5
Comorbidity severity Index	1.1 ± 0.2 [0.7–1.4]	1.1 ± 0.2 [0.7–1.6]	≤ 2
Comorbidity index	1.6 ± 0.6 [1–3]	1.8 ± 1.3 [1–4]	≤ 2
Fall risk factors	1.5 ± 1.8 [0–5]	1.5 ± 1.8 [0–5]	
Sarcopenia factors	1.9 ± 1.4 [0–4]	1.8 ± 1.6 [0–4]	
**Physical performance**			
SPPB	8.6 ± 3.3 [0–12]	7.6 ± 4.5 [1–12]	> 6
ETGUG (seconds)	33.8 ± 17.2 [22.4–86.1]	30.2 ± 14.0 [16.4–52.9] *(n = 10)*	<34
POMA	23.6 ± 4.4 [12–28]	23.6 ± 4.8 [14–28]	> 19
Steps/day	4,916 ± 2,741 [1,242–11,127] *(n = 12)*	5,878 ± 3,957 [1,815–15,862] *(n = 10)*	> 5,000
Gait speed (m/s)	0.87 ± 0.28 [0.35–1.20]	1.00 ± 0.37 [0.49–1.54] *(n = 10)*	≥ 0.80
Handgrip (kg)			
- Men	20.7 ± 7.3 [10–32]	27.2 ± 8.1 [20–40]	≥ 27
- Female	12.6 ± 3.5 [8–18]	10.8 ± 1.5 [9–13]	≥ 16
Hip flexion (kg)			
- Men	13.7 ± 3.0 [9.3–17.6]	25.0 ± 10.0 [18.0–42.4]	> 11
- Female	15.1 ± 4.4 [8.1–20.9]	12.5 ± 3.0 [8.4–17.4]	> 10
**Cognitive status**			
FAB	1.6 ± 1.2 [0–4]	1.3 ± 1.7 [0–4]	≥ 1
MMSE	26.8 ± 2.1 [22–30]	25.6 ± 4.8 [16–30]	> 24
TMT_A	1.9 ± 1.8 [0–4] *(n = 13)*	1.5 ± 1.4 [0–4] *(n = 10)*	≥ 1
TMT_B	1.7 ± 1.9 [0–4] *(n = 12)*	1.6 ± 1.9 [0–4] *(n = 9)*	≥ 1
DT cost of gait speed (%)	19.0 ± 10.0 [3–35]	18.0 ± 11.0 [−1–31] *(n = 10)*	≤ 10
**Hematology parameters**			
Calcium (mmol/l)	2.2 ± 0.2 [2–2]	2.3 ± 0.2 [2–3] *(n = 9)*	2.15–2.55
Phosphates (mmol/l)	1.54 ± 0.32 [1.02–2.19]	1.39 ± 0.26 [1.03–1.91] *(n = 9)*	0.81–1.45
Albumin (g/l)	34.6 ± 6.8 [21–45]	38.0 ± 4.4 [32–44] *(n = 8)*	35–52
CK (U/l)	151.8 ± 146.7 [36–461] *(n = 8)*	145.8 ± 65.3 [37–205] *(n = 5)*	<190
CRP (mg/l)	18.9 ± 31.2 [1–109]	6.0 ± 7.8 [1–26] (n = 9)	<5
Hemoglobin (g/l)	100.6 ± 12.8 [79–118]	108.9 ± 18.3 [84–146]	140–180
Hematocrit (L/l)	0.31 ± 0.04 [0.24–0.37]	0.33 ± 0.05 [0.26–0.43]	0.45–0.55
Ferritin (μg/l)	252.2 ± 340.3 [16.0–1,337]	390.2 ± 744.3 [0–2,218] *(n = 8)*	30–400
iPTH (pmol/l)	35.4 ± 21.3 [23.0–60.0] *(n = 3)*	167.6 ± 37.0 [7.0–1007.0] *(n = 7)*	1.6–6.7

*Where not otherwise specified, data applies to the whole group*.

On average HD started 10.1 [0–38] weeks after baseline assessment.

By comparing participants who died within 2 years after the first HD with those who survived the first 2 years, we noticed that the survivors generally had a better health status and a better performance level (see Supplementary Material S2).

Three participants who were prepared to enter HD stabilized their kidney function and did not start HD. In comparison to the participants that started HD, they were generally fitter (see Supplementary Material S2).

All baseline data are presented in Table 1.

### Evolution of Fall-Related Factors From Predialysis to 2 Years of HD

During the analyzed period, no striking changes were observed in the different parameters measured. Only SPPB (*p* < 0.01, *F* = 8.50) and numbers of comorbidities (*p* = 0.01, *F* = 3.94) show a statistically significant deterioration.

Through a nearer look at the data summarized in Table 2, we do not notice a clear tendency. If on one side some performance parameters seem to have the same evolution as the SPPB (POMA and leg strength), others do not (ETGUG, gait speed, physical activity, and handgrip). The same is observed for cognitive performance with slow deterioration in global cognition and a stable situation in executive functions and attention. The health status increases for both mental and physical health. A decrease in pain level, fatigue perception, and slightly deteriorated comorbidities also take place. EADS and ADL showed a tendency toward greater dependence and lower autonomy. HD seemed to have a negative influence on depression, but only in the first months of therapy. The results for the individual participants are collated in graphical form in Supplementary Material S3.

**Table 2:** Evolution of the studied population (mean ± SD).

	**Baseline**	**3 m**	**6 m**	**12 m**	**24 m**	**Sig**.
	**(−10.1 ±10.3 weeks)**	**(16.2 ±3.2 weeks)**	**(34.6 ±7.4 weeks)**	**(60.6 ±7.4 weeks)**	**(117.7 ±14.5 weeks)**	**p (F)**
**Health status**						
Physical health	36.5 ± 6.1	36.6 ± 8.2	34.6 ± 10.3	35.0 ± 11.6	37.0 ± 9.6	0.78 (0.34)
Mental health	49.3 ± 9.5	55.9 ± 8.4	52.8 ± 10.4	54.4 ± 9.1	53.8 ± 6.7	0.10 (2.37)
Autonomy	96.4 ± 5.0	93.9 ± 8.6	94.3 ± 8.7	94.4 ± 6.3	93.2 ± 10.8	0.32 (1.16)
Independence	15.6 ± 3.4	15.5 ± 4.4	15.0 ± 4.0	15.1 ± 4.3	13.9 ± 4.6	0.26 (1.41)
General fatigue	12.3 ± 3.4	10.9 ± 4.3	11.6 ± 3.7	11.0 ± 4.0	11.6 ± 3.6	0.68 (0.46)
Pain	42.5 ± 24.9	34.6 ± 26.1	43.2 ± 16.2	33.9 ± 21.6	29.6 ± 27.7	0.32 (1.19)
GDS-10	2.9 ± 2.2	2.4 ± 1.4	3.6 ± 1.7	3.4 ± 2.0	2.9 ± 2.1	0.32 (1.20)
Comorbidity severity index	1.1 ± 0.5	1.1 ± 0.5	1.2 ± 0.5	1.2 ± 0.5	1.2 ± 0.5	0.15 (2.09)
Comorbidity index	1.6 ± 0.7	1.8 ± 0.7	1.8 ± 0.7	1.9 ± 0.8	2.0 ± 1.0	**< 0.01 (8.50)**
Fall risk factors	1.5 ± 1.8	1.2 ± 1.7	1.8 ± 1.8	1.4 ± 1.7	1.7 ± 1.9	0.13 (1.86)
Sarcopenia factors	1.9 ± 1.4	2.2 ± 1.4	2.1 ± 1.4	2.2 ± 1.6	2.4 ± 1.3	0.31 (1.24)
**Physical performance**						
SPPB	8.6 ± 3.3	8.6 ± 2.6	8.2 ± 2.9 *(n = 13)*	8.2 ± 2.7 *(n = 13)*	7.3 ± 2.5 *(n = 13)*	**0.01 (3.94)**
ETGUG (seconds)	33.8 ± 17.2	31.6 ± 11.8	32.8 ± 14.6 *(n = 13)*	31.4 ± 11.7 *(n = 13)*	33.3 ± 13.4 *(n = 13)*	0.45 (0.75)
POMA	23.6 ± 4.4	23.6 ± 3.7	23.4 ± 4.3 *(n = 13)*	22.9 ± 3.2 *(n = 13)*	22.4 ± 5.0	0.57 (0.61)
Steps/day	4,916 ± 2,740 *(n = 12)*	4,500 ± 2,998	4,930 ± 3,065 *(n = 13)*	5,239 ± 4,488 *(n = 12)*	4,209 ± 3,296 *(n = 12)*	0.35 (1.14)
Gait speed (m/s)	0.87 ± 0.28	0.88 ± 0.28	0.86 ± 0.31 *(n = 13)*	0.93 ± 0.24 *(n = 13)*	0.87 ± 0.23 *(n = 13)*	0.49 (0.67)
Handgrip (kg)						
- Men	20.7 ± 7.3	19.9 ± 4.3	21.4 ± 4.6	21.4 ± 6.1	25.0 ± 7.6 (*n = 6)*	0.15 (2.20)
- Female	12.6 ± 3.5	12.1 ± 3.8	11.2 ± 3.7 *(n = 6)*	12.2 ± 1.8 *(n = 6)*	12.0 ± 2.9	0.83 (0.27)
Hip flexion (kg)
- Men	13.7 ± 3.0	13.4 ± 3.8	14.2 ± 3.8	13.1 ± 4.0	11.5 ± 4.1 (*n = 6)*	0.13 (2.55)
- Female	15.1 ± 4.4	13.7 ± 3.4	13.5 ± 4.2 *(n = 6)*	13.4 ± 3.4 *(n = 6)*	11.1 ± 2.7	0.24 (1.63)
**Cognitive status**						
FAB	1.6 ± 1.2	-	-	1.4 ± 1.6	1.6 ± 1.5	0.86 (0.15)
MMSE	26.8 ± 2.1	-	-	26.4 ± 2.4	26.1 ± 3.4	0.59 (0.51)
TMT_A	1.9 ± 1.8 *(n = 13)*	-	-	2.7 ± 1.6 *(n = 12)*	2.2 ± 1.8 *(n = 12)*	0.30 (1.26)
TMT_B	1.7 ± 1.9 *(n = 12)*	-	-	1.9 ± 1.8 *(n = 10)*	2.0 ± 2.0 *(n = 11)*	0.32 (1.19)
DT cost of gait speed (%)	19.1 ± 9.7	17.0 ± 8.3	18.7 ± 12.0 *(n = 13)*	16.1 ± 11.0 *(n = 13)*	17.0 ± 11.0 *(n = 13)*	0.53 (0.66)
**Hematology parameters**						
Calcium (μmol/l)	2.2 ± 0.2	2.2 ± 0.1	2.2 ± 0.1	2.2 ± 0.1	2.2 ± 0.2	0.68 (0.42)
Phosphates (μmol/l)	1.5 ± 0.3	1.4 ± 0.6	1.5 ± 0.4	1.5 ± 0.6	1.3 ± 0.6	0.34 (1.17)
Albumin (g/l)	34.6 ± 6.8	4.0 ± 4	37.6 ± 4.7	39.0 ± 39.0	36.6 ± 4.0	0.08 (2.55)
CK (U/l)	151.8 ± 146.7 *(n = 8)*	86.1 ± 59.1 *(n = 7)*	108.4 ± 36.9 *(n = 5)*	88.1 ± 32.7 *(n = 7)*	192.5 ± 331.3 *(n = 8)*	0.36 (1.10)
CRP (mg/l)	18.9 ± 31.2	15.2 ± 32.5	15.9 ± 21.4	16.1 ± 24.8	11.9 ± 11.0	0.91 (0.15)
Hemoglobin (g/l)	100.2 ± 12.4	108.4 ± 10.6	107.8 ± 9.9	109.9 ± 5.9	108.4 ± 9.6	0.10 (2.21)
Hematocrit (g/l)	0.31 ± 0.04	0.33 ± 0.03	0.33 ± 0.03	0.34 ± 0.02	0.33 ± 0.03	0.16 (1.78)
Ferritin (μg/l)	252.2 ± 340.3	418.5 ± 402.9	404.1 ± 205.7	465.0 ± 352.8	463.6 ± 354.9	0.29 (1.28)
iPTH (pmol/l)	41.6 ± 26.0 *(n = 2)*	33.3 ± 9.9 *(n = 3)*	57.3 ± 31.3 *(n = 7)*	50.7 ± 35.8 *(n = 10)*	42.1 ± 29.5 *(n = 10)*	0.36 (2.45)

*Where not otherwise specified the data applies to all participants (n = 14). The bold values signify those levels of significance that were below the 0.05% threshold value set for significance*.

Calcium, phosphates, albumin, and CRP remained rather stable, whereas CK and iPTH showed large variations throughout the period of analysis. Hemoglobin, hematocrit, and ferritin were low at baseline, increased till the first follow-up, and then stabilized by the end of the study.

All longitudinal data are summarized in Table 2.

### Adverse Events

Adverse events and falls were registered throughout the study period, from 12 months before the baseline assessment, through to the final follow-up, 2 years after the first HD. In the 12 months before baseline, of the 25 recruited participants, 7 reported 19 falls (2 falls with injury). The 14 participants that concluded the study fell 8 times (3 falls with injury) in the first year, and 9 in the second year (1 with injury). The fall rate throughout the study period (1 year before baseline until 2 years after the first HD for a mean time of 3.66 years) of the 14 participants was 0.67 per person-year for any fall, and 0.10 for serious falls. No hospitalizations were recorded in the year before baseline visit, 14 in the first year of HD (4 with surgery) and 14 in the second (7 with surgery). Four participants were institutionalized (one before starting HD and three in the first months of HD). A further 6 adverse events were recorded in the first 12 months of HD. In the dropout group, 1 transplantation and 4 deaths caused the interruption of the study in 5 participants. Compared to the participants who concluded the study, dropouts showed a higher percentage of adverse events after the first HD. Adverse events are summarized in Table 3.

**Table 3:** Adverse events.

**Adverse event**	**12 months before baseline**		Baseline^*^-first HD		**First HD–3 months**		**3 months–6 months**		**6 months–12 months**		**12 months–24 months**	
	**Completers**	**Dropouts**	**Completers**	**Dropouts**	**Completers**	**Dropouts**	**Completers**	**Dropouts**	**Completers**	**Dropouts**	**Completers**	**Dropouts**
	**(*n* = 14)**	**(*n* = 11)**	**(*n* = 14)**	**(*n* = 9)**	**(*n* = 14)**	**(*n* = 6)**	**(*n* = 14)**	**(*n* = 6)**	**(*n* = 14)**	**(*n* = 5)**	**(*n* = 14)**	**(*n* = 4)**
Falls no injury	13 (*n* = 4)	4 (*n* = 3)	3 (*n* = 2)	-	1	3 (*n* = 2)	3 (*n* = 3)	3 (*n* = 2)	1	2 (*n* = 2)	8 (*n* = 4)	1
Falls with injury	1	1	-	1	-	1	2 (*n* = 2)	-	1	5 (*n* = 3)	1	-
Hospitalisation no surgery	-	-	5 (*n* = 5)	1	4 (*n* = 4)	-	3 (*n* = 3)	3 (*n* = 2)	3 (*n* = 3)	3 (*n* = 2)	7 (*n* = 4)	-
Hospitalisation with surgery	-	-	-	2 (*n* = 2)	-	1	1	2 (*n* = 1)	3 (*n* = 2)	2 (*n* = 2)	7 (*n* = 6)	2 (*n* = 1)
Institutionalisation	1	-	-	-	-	-	1	-	-	2	-	-
Transplantation	-	-	-	-	-	-	-	-	-	-	-	1 (*n* = 1)
Death	-	-	-	-	-	-	-	1	-	1	-	2
Others	-	-	-	-	1^a^	-	4^b^ (*n* = 4)	-	1^c^	-	-	1^d^

* *in average 10 weeks before first HD*.

a *Gout in the feet, needing a wheelchair*.

b *Depressive symptoms, arm hemorrhage, Herpes Zoster, gout in the knee (needs a wheelchair)*.

c *Depressive symptoms*.

d *COVID-19*.

### *Post-hoc* Analysis

**Figure 2:**
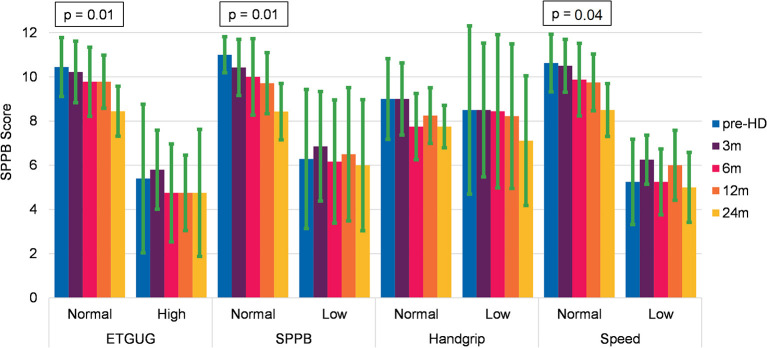
Evolution of the Short Physical Performance Battery (SPPB) in relation to baseline characteristics of physical performance tests (mean and SD).

In a *post-hoc* analysis, we investigated which baseline parameter could possibly predict a decline in physical performance in the first 2 years of HD. We compared participants with a normal score in physical performance tests (ETGUG < 34 s, SPPB ≥ 10 points, handgrip over the level defined for discriminating for sarcopenia (40), and gait speed ≥ 0.8 m/s) and those with the subnormal score. None of these parameters could predict the functional decline of POMA, gait speed, and ETGUG. The only significant decline was seen in SPPB in participants with normal performance in ETGUG (*p* = 0.01, *F* = 4.6), SPPB (*p* = 0.01, *F* = 6.04) and gait speed (*p* = 0.04, *F* = 3.6). The results are represented graphically in Figure 2.

**Figure 3:**
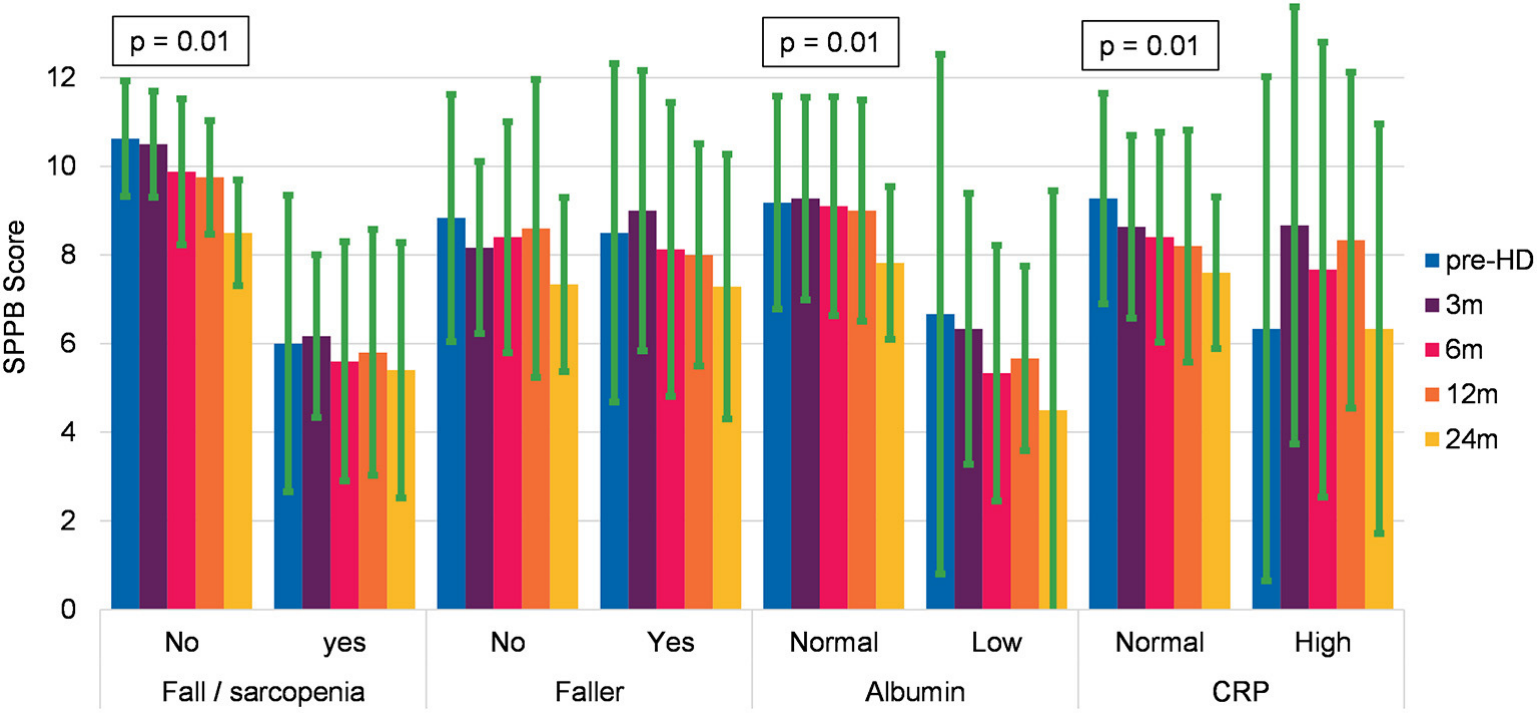
Evolution of the SPPB in relation to frailty and inflammatory status at baseline (mean and SD).

In a second step, we compared the evolution of physical performance between faller and no-faller, between those with > 1 sarcopenia/fall risk factors vs. ≤ 1 indicator, and patients with the normal inflammatory indicator (CRP ≤ 30 mg/l and albumin ≥ 30 g/l), and those with subnormal value. In this regard, POMA, ETGUG, and gait speed did not show significant decrease for any group, whereas SPPB showed a significant decrease in patients with low sarcopenia or fall risk (*p* = 0.01, *F* = 4.71), normal albumin value (*p* = 0.01, *F* = 4.3), and normal CRP value (*p* = 0.01, *F* = 4.42). The results are represented graphically in Figure 3. A detailed analysis of the data shows that the decline was significant only in the second year of HD. The results of all the groups for all the performance tests are collated in Supplementary Table S4.

## Discussion

The frailty status of the HD population is broadly described in the literature (7, 16, 84), and recently many studies have analyzed the health status of the CKD population classified into different disease stages, and thus the relationship between CKD severity and frailty (29, 85–88), slower gait speed (26), and decline in cognitive function (89) is becoming more evident. However, thus far the role of the transitional phase from predialysis to long-term dialysis in the frailty development process of patients with CKD is not well understood (32). The goal of our study was to clarify the role of HD initiation in the development of frailty in the ESRD population. We hypothesized that the frailty seen in the patients having HD is neither directly (due to the dialysis session) nor indirectly (e.g., reduced physical activity) caused by the replacement therapy, but is rather preexistent. During the study planning, many studies were focusing on patients with HD, but little was known about the early stages of CKD.

The baseline characteristics of the participants show a rather heterogenous population, ranging between young seniors (< 65 years old) engaging regularly in activities such as mountain-trekking (gait speed of 1.54 m/s), to old seniors (> 80 years old) living in a nursing home and needing a walker to support their mobility (gait speed of 0.35 m/s). Most of the participants had functional abilities lying somewhere between these two extremes. The following baseline characteristics of our sample do not differ significantly from the HD population described in the literature: health status (90–92), fatigue (93), autonomy (16, 94), physical functioning (23, 26, 95–97), cognition (29, 98, 99), fall rate (30), and hematology parameters (100–102). Our sample was better in pain (103), depression syndrome (104), physical activity level (105, 106), and comorbidities. However, this could be due to the definition we adopted, which only considers comorbidities classified as “*severe and constant significant disabling or uncontrollable chronic problems”* (77). Handgrip was the only parameter found to be lower than in the HD population (107, 108) and below the cut-off score for sarcopenia (40). All the participants were in a predialysis program, and were, therefore, regularly checked by the nephrologist. In this way, health status could be monitored, and comorbidities were taken into consideration throughout the years. Baseline assessments were based on clinical decisions made at the moment when the uremic curve was indicating a clear tendency toward kidney failure. At this point, most participants did not have acute symptoms of reduced kidney function, e.g., fatigue, shortness of breath, confusion, weakness, fluid retention, or confusion (109), and dialysis began before the symptoms arose. The transition from predialysis to dialysis was gradually planned. This could explain the absence of commonly described problems such as insomnia, restless leg syndrome and uremic pruritus (110)—only one patient developed a problem directly related to HD, namely an allergic reaction to the filter—and why the health status of the participants did not deteriorate abruptly. Our results seem to confirm the importance of a predialysis program (111).

Comparing participants with normal health characteristics (defined as fit) to those with frailty indicators, revealed some interesting aspects regarding the evolution of the physical performance as measured by the SPPB (Figures 2, 3). Combining the subgroup analysis with the analysis of the dropouts, we observe the following tendency: fit patients in the predialysis stage can stabilize their kidney function and postpone HD start. Once they start HD, their physical performance remains quite stable in the first year and then starts to deteriorate. This finding suggests the importance to monitor patients new to HD not only in the first year but also in the following period. Frail people who start HD remain quite stable in their frailty level, but an adverse event can rapidly affect their health status and lead to death. The SPPB—a widely used assessment that is predictive for many outcomes like falls, institutionalization, and death in elderly people (112)—is validated for the CKD population with the minimal detectable difference of 1.7 points indicating a real decline in physical performance (96). In our study, SPPB seems to be the only measure sensitive to changes during the first 2 years of HD. Though this finding has to be treated with prudence due to the small sample size and due to the fact that other relevant indicators for health status (ETGUG, handgrip, and gait speed) remained stable.

The stability in the health of fit patients and the low mortality in frail participants (113) may confirm the effectiveness of a predialysis program. On the other hand, the high mortality in frail people and the decline in physical performance in the second year of HD could be a justification for considering introducing frailty prevention programs for both frail and fit patients new to HD, to prevent long-term decline that in frail people can rapidly result in a terminal outcome.

### Strength and Limitations

Although our study is one of the first to apply a longitudinal study design with the aim of monitoring functioning post-HD, several limitations need to be mentioned. Since this study was designed as a pilot observational study, it consequently has a small sample size. All the conclusions, therefore, need to be treated as exploratory in nature. Future research should consider and include information on the health status of a larger CKD individuals' sample on the national level. Patients were referred to this study at the discretion of their treating physician and it, therefore, remains uncertain if the small sample represents the same properties and proportions as that of a larger population. However, the nature of the study was decided after reviewing the literature and finding no previous study on this important transition. Therefore, even this limited range of patients, comprehensively and longitudinally assessed, is an important step forward in our understanding.

Considering the high variability in functioning we observed in our small sample, we are confident our data is representative of this population. The inclusion of patients that had to be in a predialysis program is a possible further limitation. This is because the design of the study leads to a selection bias, and our conclusions cannot, therefore, be transferred to patients who do not follow such a program.

Compared with other studies of patients with HD, the mean age of our sample was higher, and this may have confounded the number of frailty cases since many factors are related to age.

## Conclusion

The role of HD initiation in the frailty process of patients with CKD remains controversial. We intended to describe the transitional phase from predialysis through 2 years of HD in a multifactorial way. We found a highly heterogenous population at baseline, with a majority of the included frail individuals not showing a significant decrease in health status during the analyzed period. However, a detailed analysis may reveal the important role of a predialysis program that permits a more acceptable transition into HD, both physical and psychological. The subgroup analysis shows the importance of health status in the predialysis stage, especially in the long term. If during the first year, no significant changes were registered, neither in frail nor in fit people, in the second year, frail people had a higher chance of dying and fitter people began to deteriorate physically. This may suggest the importance of a frailty prevention program even for fit patients in addition—where possible—to a predialysis program. The results warrant a larger trial performed country-wide in Switzerland. This proposed larger longitudinal study can be methodologically similar, using similar subjects, the same setting, and the same techniques of data collection and analysis, however, on a larger scale.

## Data Availability Statement

The original contributions presented in the study are included in the article/Supplementary Material, further inquiries can be directed to the corresponding author.

## Ethics Statement

The studies involving human participants were reviewed and approved by Comitato etico del Canton Ticino with ID number 2019-01161, CE 3497. The patients/participants provided their written informed consent to participate in this study. The study procedures were carried out in accordance with the 1964 Declaration of Helsinki (114) and subsequent amendments. The manuscript was created in accordance with STROBE guidelines (115, 116). The Checklist is available in Supplementary Table S5. During the pandemic period all protection policies defined by the Federal and Cantonal Health Department were respected during the visits.

## Disclosure

The results presented in this article have not been published previously in whole or in part.

## Author Contributions

DZ designed the study, collected and analyzed the data, and wrote the manuscript. OG designed the study, recruited patients, collected data, and contributed to the writing of the manuscript. PQ, MT, and EB designed the study and contributed to the writing of the manuscript. LB, SP, and CS approved the study protocol, recruited the patients, collected data, and reviewed critically the manuscript. All authors approved the final version of the manuscript.

## Funding

This study was financed by the Scientific Research Advisory Board of the Ente Ospedaliero Cantonale (ABREOC) with Grant Number 22061.

## Conflict of Interest

The authors declare that the research was conducted in the absence of any commercial or financial relationships that could be construed as a potential conflict of interest.

## Publisher's Note

All claims expressed in this article are solely those of the authors and do not necessarily represent those of their affiliated organizations, or those of the publisher, the editors and the reviewers. Any product that may be evaluated in this article, or claim that may be made by its manufacturer, is not guaranteed or endorsed by the publisher.
