# Supplementary material for: A Pilot Observational Study Assessing Long-Term Changes in Clinical Parameters, Functional Capacity and Fall Risk of Patients With Chronic Renal Disease Scheduled for Hemodialysis
Source: Front Med (Lausanne). 2022 Feb 4;9:682198. doi: 10.3389/fmed.2022.682198 (PMC8854975; doi:10.3389/fmed.2022.682198)
Supplement: Supplementary file 1 [file Data_Sheet_1.docx]

Supplementary Material

**S1: Supplementary Information**

| **Assessment** | **Abbreviation** | **Unit** | **Reference value** |
| --- | --- | --- | --- |
| **Body Mass Index** | BMI | kg/m^2^ | 18.5 – 24.9 |
| **Expanded Timed Get-up-and-Go Test** | ETGUG | s | < 34 |
| **Performance Oriented Mobility Assessment** | POMA | 0 – 28 points | > 19 |
| **Short Physical Performance Battery** | SPPB | 0 – 12 points | > 6 |
| **Handgrip** | HG | Kg | ≥ 27 (male)  ≥ 16 (female) |
| **Hip Flexion** | HF | Kg | > 11 (male)  > 10 (female) |
| **Gait speed** | - | m/s | > 0.8 |
| **Dual-task cost of gait** | DTC | % | < 10 |
| **Weekly monitoring of physical activity** | - | Steps/day | > 5’000 |
| **The Short Form health survey** | SF-12 | 24 – 57  19 – 61 points | > 40 (physical)  > 40 (mental) |
| **The Barthel Index of activities of daily living** | ADL | 0 – 100 points | ≥ 75 |
| **Nottingham Extended Activities of Daily Living Scale** | EADS | 0 – 22 points | ≥ 17 |
| **Visual Analog Scale for pain** | VAS-P | 0 – 100 points | < 40 |
| **Geriatric Depression Scale** | GDS-10 | 0 – 10 points | < 4 |
| **Multidimensional fatigue inventory** | MFI-20 | 0 – 24 points | > 10 |
| **Mini-Mental State Examination** | MMSE | 0 – 30 points | > 24 |
| **Frontal Assessment Battery** | FAB | 0 – 4 points* | ≥ 1 |
| **Trail Making Test** | TMT | 0 – 4 points* | ≥ 1 |
| **Cumulative illness rating scale** | CIRS-S  CIRS-C | 0 – 4  0 – 14 points | ≤ 2 (severity)  ≤ 2 (comorbidity) |
| **Haematology parameters**   - **Calcium** - **Phosphates** - **Albumin** - **Creatine kinase** - **C-reactive protein** - **Haemoglobin** - **Haematocrit** - **Ferritin** - **Intact parathyroid Hormone** | Ca^++^  PO_4_^-^  CK  CRP  iPTH | μmol/L  μmol/L  g/L  U/L  mg/L  g/L  g/L  μg/L  pmol/L | 2.15 – 2.55  0.81 – 1.45  35 – 52  < 190  < 5  140 – 180  0.45 – 0.55  30 – 400  1.6 – 6.69 |
| * adjusted score | | | |

Tests description

**Expanded Timed Get-up-and-Go Test (ETGUG)**

The test measures the time required to stand up, walk 10 m, turn, walk back, and sit down.

**Short Physical Performance Battery (SPPB)**

The SPPB includes tests of walking speed, standing balance and chair stand. Each SPPB component test (gait, balance and chair stand) is scored from 0 to 4, with a score of 0 representing inability to carry out the test, and 4 as the best performance. For balance, the participants are asked to maintain their feet side-by-side, in semi-tandem and tandem positions for 10 s each. For gait, a 4m walk at the participants’ usual speed was timed with a standing start. For the chair stand test, participants were asked to stand up and sit down five times as quickly as possible.

**Performance Oriented Mobility Assessment (POMA)**

POMA is a task- oriented test that measures an older adult's gait and balance abilities. Scoring of the Tinetti Assessment Tool is done on a three-point ordinal scale with a range of 0 to 2. A score of 0 represents the most impairment, while a score of 2 represents independence. The individual scores are then combined to form three measures; an overall gait assessment score, and overall balance assessment score, and a combined gait and balance score. The maximum score for the gait component is 12 points. The maximum score for the balance component is 16 points. The maximum total score is 28 points.

**Short Form health survey (SF-12)**

The, SF-12 is a commonly used instrument to measure the health-related quality of life at various ages. The instrument uses 12 questions to measure functional health and wellbeing from the patient’s perspective. The generic health-status measure reproduces the physical component summary (PCS) score and the mental component summary (MCS) score. The SF-12 is validated for long dialysis patients.

**Barthel Index of activities of daily living (ADL)**

The ADL assesses functional disability by quantifying patient performance in 10 activities of daily life. Scoring is done on a three-level scale: 0 (unable), 5 (needs help) and 10 (independent). The maximum score is 100.

**Nottingham Extended Activities of Daily Living Scale (EADS)**

The EADS is used for assessing independence and includes 4 sections (mobility, kitchen, domestic and leisure) for a total of 22 items measuring the capacity to do a specific task. A score of 0 means the person needs help and 1 means the person is independent. The maximum score is 22.

**Geriatric Depression Scale (GDS-10)**

The GDS-10 is a screening test for depression in the elderly population, but validated also for younger adults. The scale is a 10-item, self-reporting instrument that uses Yes (1 points) and No (0 points) answers. A total score of 0 means no signs of depression.

**Multidimensional fatigue inventory (MFI-20)**

The MFI-20 is a 20-item scale designed to evaluate five dimensions of fatigue: general fatigue, physical fatigue, reduced motivation, reduced activity, and mental fatigue. The instrument includes 20 statements that are evaluated on a 5-point Likert Score scale (1 = I completely agree, 5 = I don’t agree at all).

**Minimental State Examination (MMSE)**

The MMSE is a neuropsychological test that assess cognitive disorders. It’s a widely used tool that permits a first evaluation of a patients but also to follow the evolution of the cognitive disorders in time. It contains tasks of spatial and temporal orientation, word registration and recall, attention and calculation, language skill, repetition and complex commands that are scored 0 to 30 points.

**Frontal Assessment Battery (FAB)**

The FAB is a short cognitive and behavioural six-subtest battery for the screening of a global executive dysfunction. The global performance on these six subtests (conceptualization, mental flexibility, motor programming, sensitivity to interference, inhibitory control and environmental autonomy) gives a composite score summarizing the severity of the dysexecutive syndrome.

**Trail Making Test (TMT)**

The TMT is a neuropsychological test of visual attention and task switching. It consists of two parts (TMT-A and TMT-B), in which the subject is instructed to connect a set of 25 dots as quickly as possible while still maintaining accuracy. The test can provide information about visual search speed, scanning, speed of processing, mental flexibility, as well as executive functioning.

**Cumulative illness rating scale (CIRS)**

The CIRS measures the chronic medical illness (morbidity) burden while taking into consideration the severity of chronic diseases in 14 items representing individual body systems. The general rules for severity rating are: 0 = no problem, 1 = mild problem, 2 = requires first line therapy, 3 = severe problem, 4 = immediate treatment required.

S2: Main demographic, clinical and functional data (mean ± SD [min – max]) at baseline by groups. The results in green represent the best outcome between the groups. Where not otherwise specified, data applies to the whole group.

|  | **Completers**  **(n=14)** | | **Dropouts**  **(n=11)** | | | | **Survivors**  **(n=21)** | | | **Death**  **(n=4)** | | | | **HD**  **(n=22)** | | | **No HD**  **(n=3)** | **Reference value** |
| --- | --- | --- | --- | --- | --- | --- | --- | --- | --- | --- | --- | --- | --- | --- | --- | --- | --- | --- |
| **General characteristics** | | | | | | |  | |  | |  | |  | |  | | | |
| Gender (M / W) | 7 / 7 | | 5 / 6 | | | | 11 / 10 | | | 1 / 3 | | | | 10 / 12 | | | 2 / 1 |  |
| Age (years) | **72.4±5.4**  **[60-81]** | | 76.6±6.5  [64-86] | | | | **73.1±5.7**  **[60-82]** | | | 80±5.8  [75-86] | | | | **73.9±6.1**  **[60-86]** | | | 76.7±6.8  [69-82] |  |
| BMI (kg/m^2^) | 29.7±3.6  [22.6-35.1] | | **28.9±6.5**  **[16.7-40.2]** | | | | 29.9±5.1  [16.7-40.2] | | | **26.4±3.2**  **[22.9-30.7]** | | | | **29.1±5.0**  **[16.7-40.2]** | | | 30.9±4.8  [27.6-36.4] | 18.5 – 24.9 |
| Schooling  (years) | 8.2±3.3  [5-17] | | 8.2±3.8  [3-17] | | | | **8.5±3.5**  **[5-17]** | | | 6.5±2.4  [3-8] | | | | 7.6±2.9  [3-17] | | | **12.3±4.5**  **[8-17]** |  |
| Household (alone / with others) | 6 / 8 | | 4 / 7 | | | | 8 / 13 | | | 2 / 2 | | | | 9 / 13 | | | 1 / 2 |  |
| **Health status** |  | |  | | | |  | | |  | | | |  | | |  |  |
| Physical health | 36.5±5.9  [25.0-49.6] | | **40.3±10.0**  **[23.4-54.2]** | | | | **38.8±8.1**  **[23.4-54.2]** | | | 34.8±7.8  [24.2-42.9] | | | | 36.9±7.4  [23.4-52.5] | | | **47.5±6.5**  **[41.3-54.2]** | > 40 |
| Mental health | 49.3±9.6  [28.3-65.1] | | **54.7±8.1**  **[34.1-63.6]** | | | | 51.6±10  [28.3-65.1] | | | **52±1.6**  **[50.8-54.1]** | | | | 51.6±8.5  [28.3-65.1] | | | **52.2±15.8**  **[34.1-63.6]** | > 40 |
| Autonomy | 96.4±5  [85-100] | | **97.3±5.2**  **[85-100]** | | | | **97.6±4.4**  **[85-100]** | | | 92.5±6.5  [85-100] | | | | 96.4±5.2  [85-100] | | | **100±0**  **[100-100]** | ≥ 75 |
| Independence | 15.6±3.4  [9-20] | | **17.0±3.3**  **[12-22]** | | | | **16.4±3.4**  **[9-22]** | | | 15.5±3  [12-18] | | | | 15.9±3.3  [9-22] | | | **19.0±1.7**  **[18-21]** | ≥ 17 |
| General fatigue | **12.3±3.4**  **[8-19]** | | 10.8±4.4  [5-17] | | | | 11.1±4.1  [5-19] | | | **14.3±1**  **[13-15]** | | | | **12.0±3.4**  **[7-19]** | | | 9.0±6.9  [5-17] | > 10 |
| Pain | 42.5±24.9  [5-100] | | **26.8±29.9**  **[0-100]** | | | | **33.8±25.8**  **[0-100]** | | | 45±39.8  [5-100] | | | | 37.5±28.4  [0-100] | | | **21.7±20.8**  **[5-45]** | < 40 |
| GDS-10 | **2.9±2.2**  **[0-7]** | | 3.4±3.3  [0-10] | | | | **2.7±2.5**  **[0-7]** | | | 5.3±3.2  [3-10] | | | | 3.2±2.6  [0-10] | | | **2.3±4.0**  **[0-7]** | ≤ 5 |
| Comorbidity severity Index | 1.1±0.2  [0.7-1.4] | | 1.1±0.2  [0.7-1.6] | | | | **1.1±0.2**  **[0.7-1.4]** | | | 1.2±0.3  [1-1.6] | | | | 1.1±0.2  [0.7-1.6] | | | **0.8±0.1**  **[0.7-0.9]** | ≤ 2 |
| Comorbidity index | **1.6±0.6**  **[1-3]** | | 1.8±1.3  [1-4] | | | | **1.5±0.7**  **[1-3]** | | | 2.5±1.7  [1-4] | | | | 1.8±1.0  [1-4] | | | **1.0±0.0**  **[1-1]** | ≤ 2 |
| Fall risk factors | 1.5±1.8  [0-5] | | 1.5±1.8  [0-5] | | | | **1.2±1.6**  **[0-5]** | | | 3.0±2.2  [0-5] | | | | 1.6±1.8  [0-5] | | | **0.3±0.6**  **[0-1]** |  |
| Sarcopenia factors | 1.9±1.4  [0-4] | | **1.8±1.6**  **[0-4]** | | | | **1.6±1.3**  **[0-4]** | | | 3.3±1.5  [1-4] | | | | 2.0±1.5  [0-4] | | | **1.0±0.0**  **[1-1]** |  |
| **Comorbidities** | | | |  |  | |  | | |  | | | |  | | |  |  |
| - *Heart* | | 2 | - | | | | 3 | | | 1 | | | | 4 | | | - |  |
| - *Vascular* | | 1 | - | | | | 1 | | | - | | | | 1 | | | - |  |
| - *Hematopoietic* | | 1 | 3 | | | | 2 | | | 2 | | | | 4 | | | - |  |
| - *Respiratory* | | 3 | 1 | | | | 4 | | | - | | | | 4 | | | - |  |
| - *Eyes, ears, nose, throat, larynx* | | - | - | | | | - | | | - | | | | - | | | - |  |
| - *Upper gastrointestinal tract* | | - | - | | | | - | | | - | | | | - | | | - |  |
| - *Lower gastrointestinal tract* | | - | 1 | | | | 4 | | | 1 | | | | 1 | | | - |  |
| - *Liver, pancreas and biliary* | | - | 1 | | | | - | | | 1 | | | | 1 | | | - |  |
| - *Renal* | | 14 | 11 | | | | 21 | | | 4 | | | | 22 | | | 3 |  |
| - *Genitourinary* | | 1 | - | | | | 1 | | | - | | | | 1 | | | - |  |
| - *Musculoskeletal and skin* | | - | - | | | | - | | | - | | | | - | | | - |  |
| - *Neurologic* | | - | - | | | | - | | | - | | | | - | | | - |  |
| - *Endocrine and breast* | | 2 | 1 | | | | 2 | | | 1 | | | | 3 | | | - |  |
| - *Psychiatric illness* | | - | - | | | | - | | | - | | | | - | | | - |  |
| **Physical Performance** | | |  | | | |  | |  | | |  | | | |  | | |
| SPPB | **8.6±3.3**  **[0-12]** | | 7.6±4.5  [1-12] | | | | **9.0±3.3**  **[0-12]** | | | 4.3±4  [1-10] | | | | 7.9±3.9  [0-12] | | | **10.3±2.1**  **[8-12]** | > 6 |
| ETGUG  (seconds) | 33.8±17.2  [22.4-86.1] | | **30.2±14.0**  **[16.4-52.9]**  ***(n=10)*** | | | | **30±15.6**  **[16.4-86.1]**  ***(n=20)*** | | | 43.6±12.3  [26.3-52.9] | | | | 33.5±16.4  [16.4-86.1]  *(n=21)* | | | **23.8±4.4**  **[18.7-26.6]** | > 34 |
| POMA | 23.6±4.4  [12-28] | | 23.6±4.8  [14-28] | | | | **24.4±4**  **[12-28]** | | | 19.3±4.8  [14-25] | | | | 23.0±4.5  [12-28] | | | **27.3±1.2**  **[26-28]** | > 19 |
| Steps/day | 4916±2741  [1242-11127]  *(n=12)* | | **5878±3957**  **[1815-15862]**  ***(n=10)*** | | | | **5720±3517**  **[1242-15862]** | | | 3700±1284  [1815-4677] | | | | 5252±3490  [1242-15862]  *(n=19)* | | | **5993±1977**  **[4071-8022]** | > 5000 |
| Gait speed  (m/s) | 0.87±0.28  [0.35-1.20] | | **1.00±0.37**  **[0.49-1.54]**  ***(n=10)*** | | | | **0.97±0.32**  **[0.35-1.54]**  ***(n=20)*** | | | 0.69±0.24  [0.49-1.03] | | | | 0.90±0.33  [0.35-1.54]  *(n=21)* | | | **1.08±0.26**  **[0.84-1.35]** | ≥ 0.80 |
| Handgrip (kg)   - Men - Female | 20.7±7.3  [10-32]  **12.6±3.5**  **[8-18]** | | **27.2±8.1**  **[20-40]**  10.8±1.5  [9-13] | | | | **23.6±2.5**  **[10-40]**  **12.1±0.9**  **[8-18]** | | | 22.0  10.7±1.2  [9-13] | | | | **23.7±2.7**  **[10-40]**  **11.9±0.8**  **[8-18]** | | | **22.0±2.0**  **[20-24]**  **20.0** | ≥ 27  ≥ 16 |
| Hip flexion (kg)   - Men - Female | 13.7±3.0  [9.3 – 17.6]  **15.1±4.4**  **[8.1 – 20.9]** | | **25.0±10.0**  **[18.0 – 42.4]**  12.5±3.0  [8.4 – 17.4] | | | | 18.4±2.7  [9.3-42.4]  **14.4±1.4**  **[8.1-20.9]** | | | **18.5**  12.2±0.90  [10.4-13.3] | | | | **18.0±3.0**  **[9.3-42.4]**  **14.0±1.2**  **[8.1-20.9]** | | | **20.5±2.5**  **[18-23]**  **23.0** | > 11  > 10 |
| **Cognitive Status** | | |  | | | |  | |  | | |  | | | |  | | |
| FAB | **1.6±1.2**  **[0-4]** | | 1.3±1.7  [0-4] | | | | **1.8±1.4**  **[0-4]** | | | 0.0±0.0  [0-0] | | | | 1.4±1.3  [0-4] | | | **2.3±2.1**  **[0-4]** | ≥ 1 |
| MMSE | **26.8±2.1**  **[22-30]** | | 25.6±4.8  [16-30] | | | | **26.7±2.9**  **[18-30]** | | | 24±5.9  [16-29] | | | | 25.9±3.6  [16-30] | | | **29±1.0**  **[28-30]** | > 24 |
| TMT_A | **1.9±1.8**  **[0-4]**  ***(n=13)*** | | 1.5±1.4  [0-4]  *(n=10)* | | | | **2.0±1.6**  **[0-4]**  ***(n=19)*** | | | 0.5±1  [0-2] | | | | **1.8±1.7**  **[0-4]**  ***(n=20)*** | | | 1.7±0.6  [1-2] | ≥ 1 |
| TMT_B | **1.7±1.9**  **[0-4]**  ***(n=12)*** | | 1.6±1.9  [0-4]  *(n=9)* | | | | **1.7±1.8**  **[0-4]**  ***(n=18)*** | | | 1.3±2.3  [0-4]  *(n=3)* | | | | 1.6±1.9  [0-4]  *(n=18)* | | | **2±1.7**  **[1-4]** | ≥ 1 |
| DT cost of gait speed (%) | 19.0±10.0  [3-35] | | **18.0±11.0**  **[-1-31]**  ***(n=10)*** | | | | **18.3±10.3**  **[-1-35]**  ***(n=20)*** | | | 21±8.8  [10-31] | | | | 19.2±10  [-1-35]  *(n=21)* | | | **15.3±10.1**  **[9-27]** | ≤ 10 |
| **Haematology parameters** | | | | | |  | |  | | | | | | | | | | |
| Calcium (mmol/L) | **2.2±0.2**  **[2-2]** | | **2.3±0.2**  **[2-3]**  ***(n=9)*** | | | | **2.2±0.2**  **[1.62-2.53]**  ***(n=19)*** | | | **2.4±0.2**  **[2.11-2.64]** | | | | 2.2±0.2  [1.62-2.64]  *(n=20)* | | | 2.4±0.2  [2.21-2.53] | 2.15 – 2.55 |
| Phosphates (mmol/L) | 1.54±0.32  [1.02-2.19] | | **1.39±0.26**  **[1.03-1.91]**  ***(n=9)*** | | | | 1.50±0.29  [1.02-2.19]  *(n=19)* | | | **1.39±0.38**  **[1.03-1.91]** | | | | 1.50±0.31  [1.02-2.19]  *(n=20)* | | | **1.35±0.19**  **[1.13-1.46]** | 0.81 – 1.45 |
| Albumin (g/L) | 34.6±6.8  [21-45] | | **38±4.4**  **[32-44]**  ***(n=8)*** | | | | **35.5±6.4**  **[21-45]**  ***(n=19)*** | | | **38±4.6**  **[33-42]**  ***(n=3)*** | | | | 35.1±6.2  [21-45]  *(n=19)* | | | 40.7±4.2  [36-44] | 35 – 52 |
| CK (U/L) | **151.8±146.7**  **[36-461]**  ***(n=8)*** | | **145.8±65.3**  **[37-205]**  ***(n=5)*** | | | | **154.6±123.2**  **[36-461]**  ***(n=11)*** | | | **121±118.8**  **[37-205]**  ***(n=2)*** | | | | **147.4±123.3**  **[36-461]**  ***(n=12)*** | | | **174.0±0.0**  **[174-174]**  ***(n=1)*** | < 190 |
| CRP (mg/L) | 18.9±31.2  [1-109] | | 6±7.8  [1-26]  (n=9) | | | | 14.8±27.4  [1-109]  *(n=19)* | | | 9.3±11.4  [1-26] | | | | 15.4±26.8  [1-109]  *(n=20)* | | | **3.7±2.5**  **[1-6]** | < 5 |
| Haemoglobin (g/L) | 100.6±12.8  [79-118] | | 108.9±18.3  [84-146] | | | | 106.2±16.1  [79-146] | | | 94.3±9.3  [84-104] | | | | 100.4±11.7  [79-118] | | | 132.7±11.5  [126-146] | 140 – 180 |
| Haematocrit (L/L) | 0.31±0.04  [0.24-0.37] | | 0.33±0.05  [0.26-0.43] | | | | 0.33±0.05  [0.24-0.43] | | | 0.29±0.03  [0.26-0.32] | | | | 0.31±0.04  [0.24-0.37] | | | 0.40±0.02  [0.39-0.43] | 0.45 – 0.55 |
| Ferritin (μg/L) | **252.2±340.3**  **[16-1337]** | | **390.2±744.3**  **[0-2218]**  ***(n=8)*** | | | | **233.4±302.9**  **[16.2-1337.4]**  ***(n=18)*** | | | 612.7±1072.3  [0.32-2218] | | | | 323.6±532.0  [0.32-2218]  *(n=20)* | | | 90.5±68.6  [42-139]  *(n=2)* | 30 – 400 |
| iPTH (pmol/L) | 35.4±21.3  [23.0-60.0]  *(n=3)* | | 167.6±371  [7.0-1007.0]  *(n=7)* | | | | 23±19.5  [7.3-60.0]  *(n=6)* | | | 285.5±481.8  [14.4-1007.0] | | | | 178.3±366.2  [14.4-1007.0]  *(n=7)* | | | 10.5±5.4  [7.3-16.8] | 1.6 – 6.7 |

**S3: Evolution of the studied population. The coloured line represent the single participant and the black lines the mean value.**

| 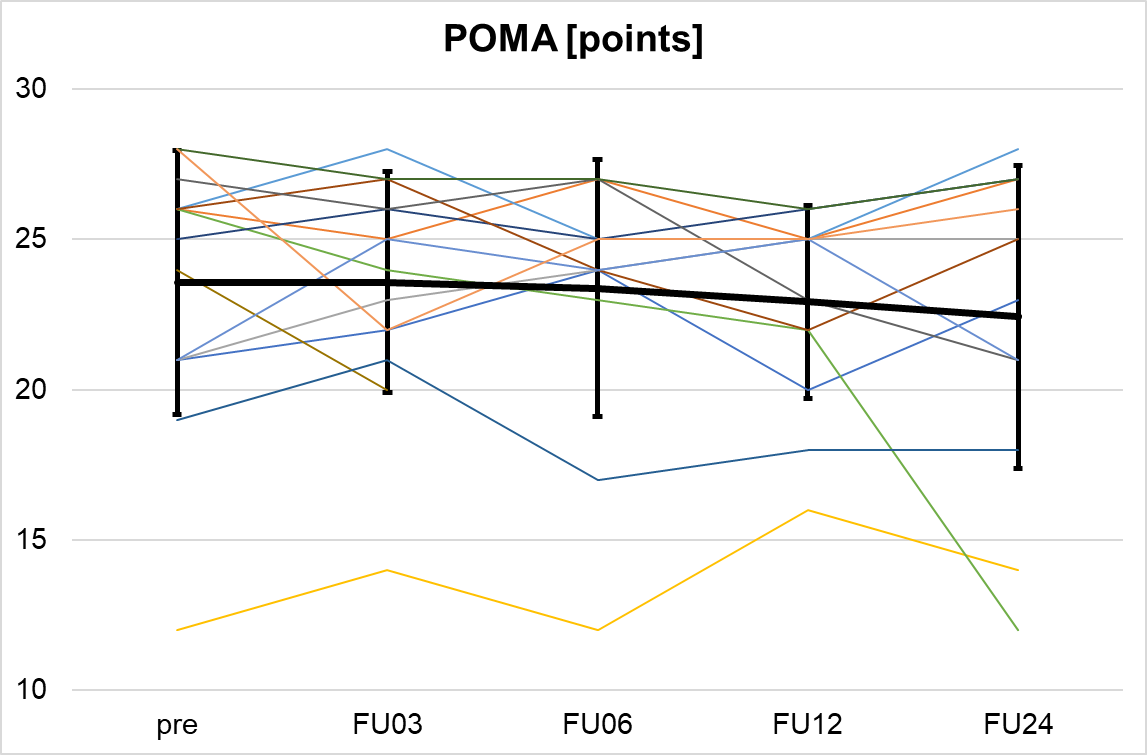 | 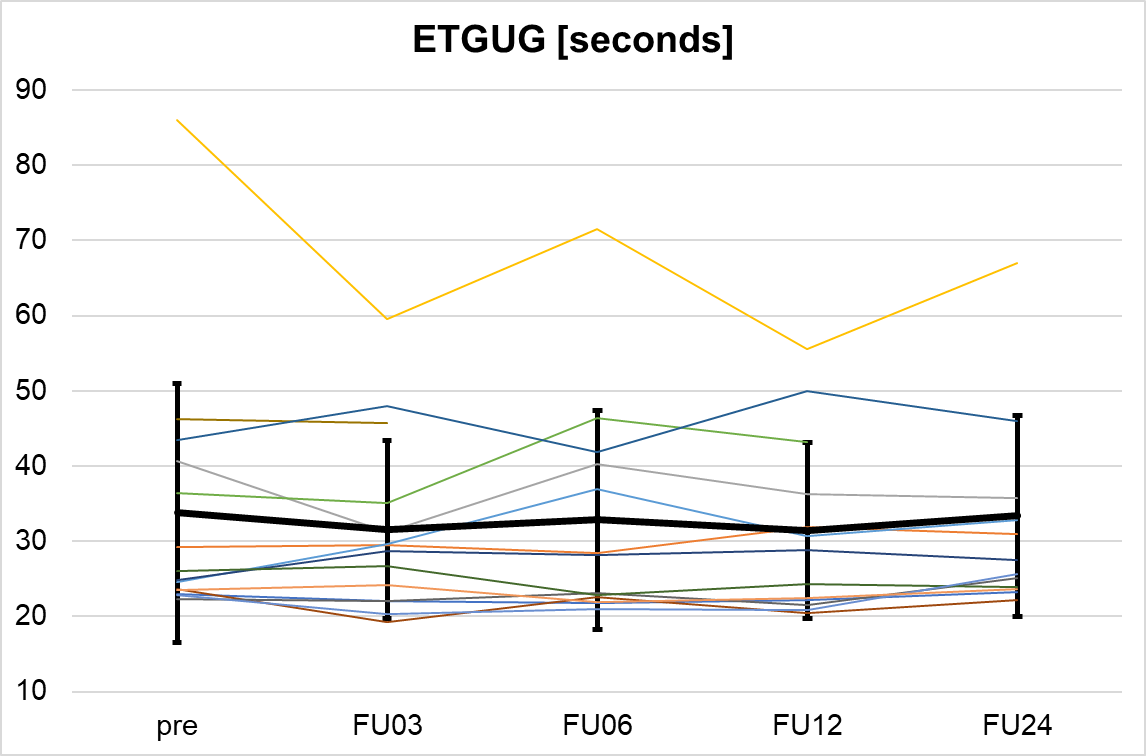 |
| --- | --- |
| 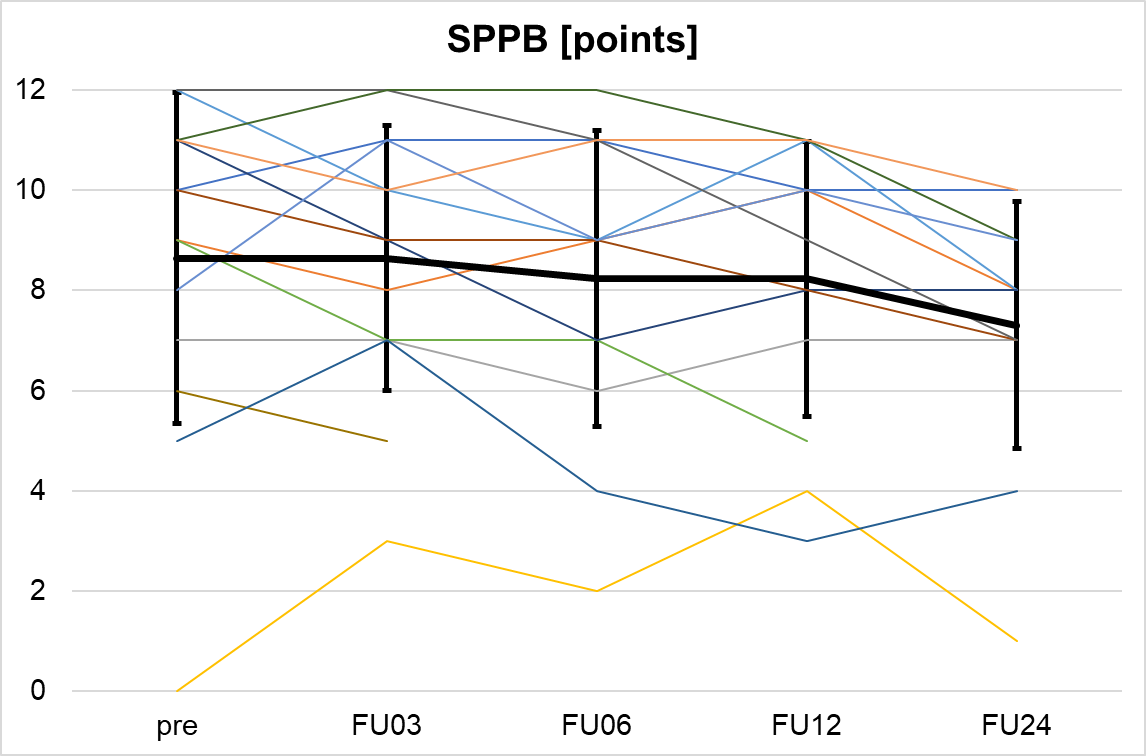 | 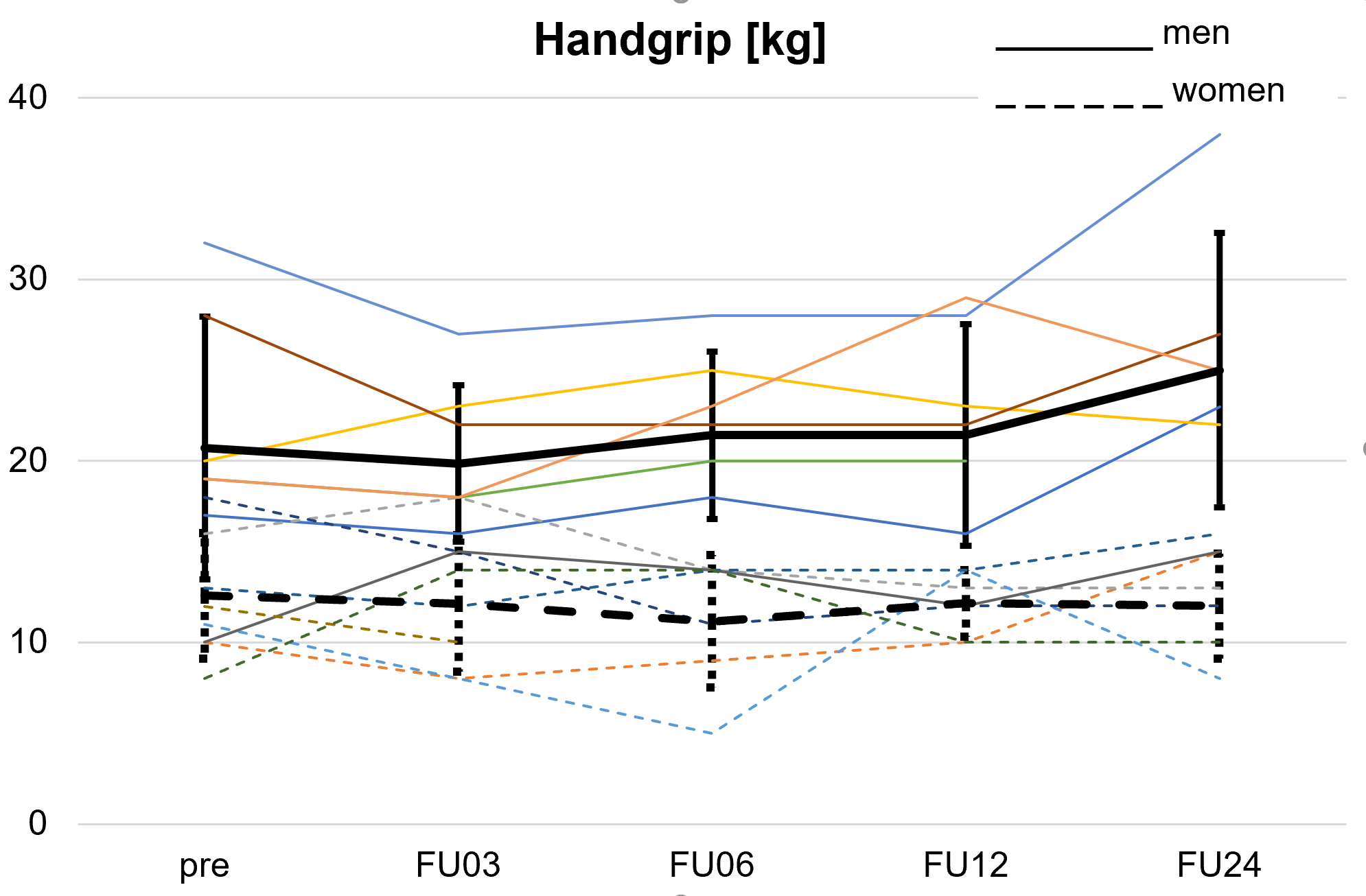 |
| 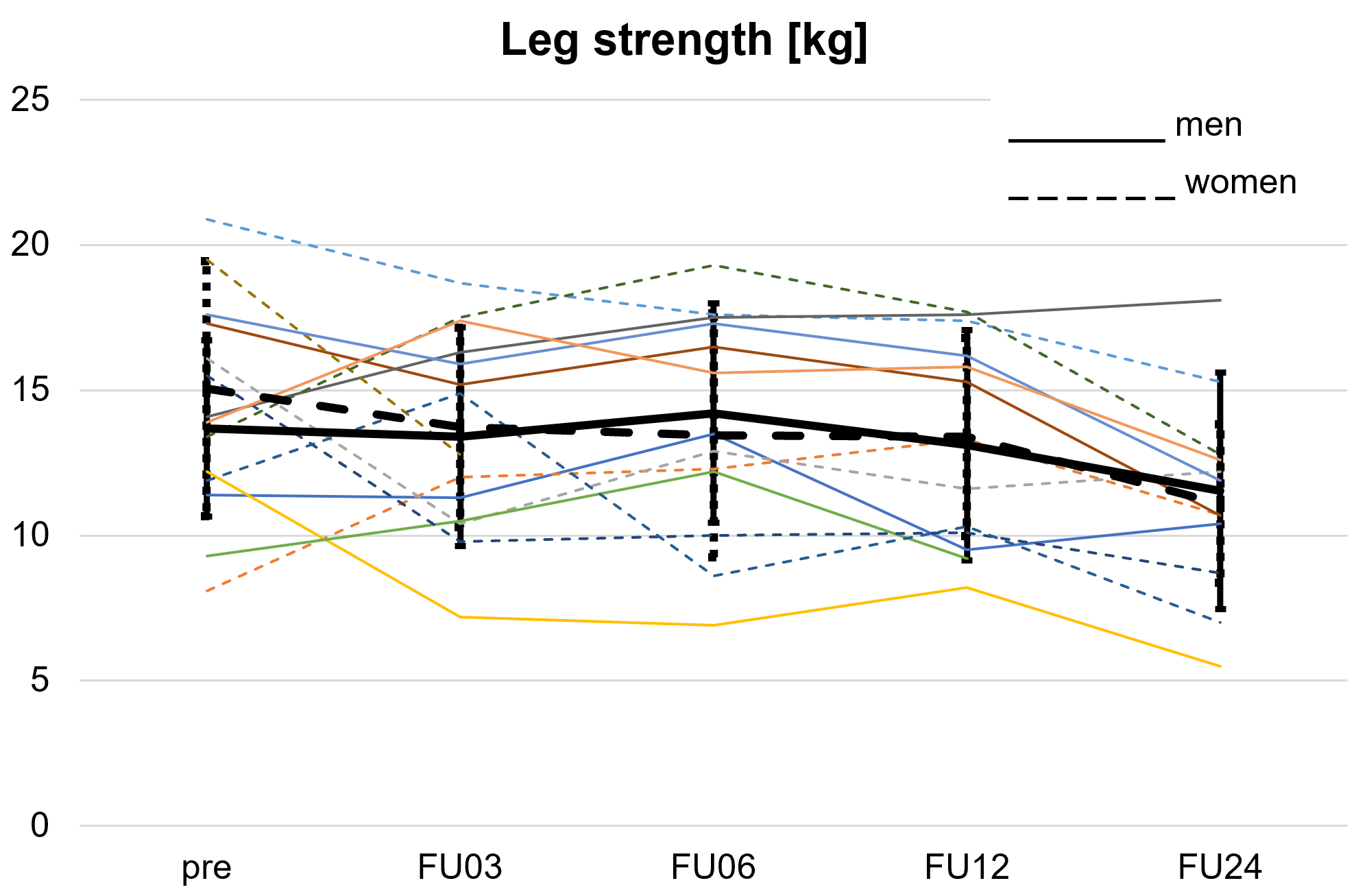 | 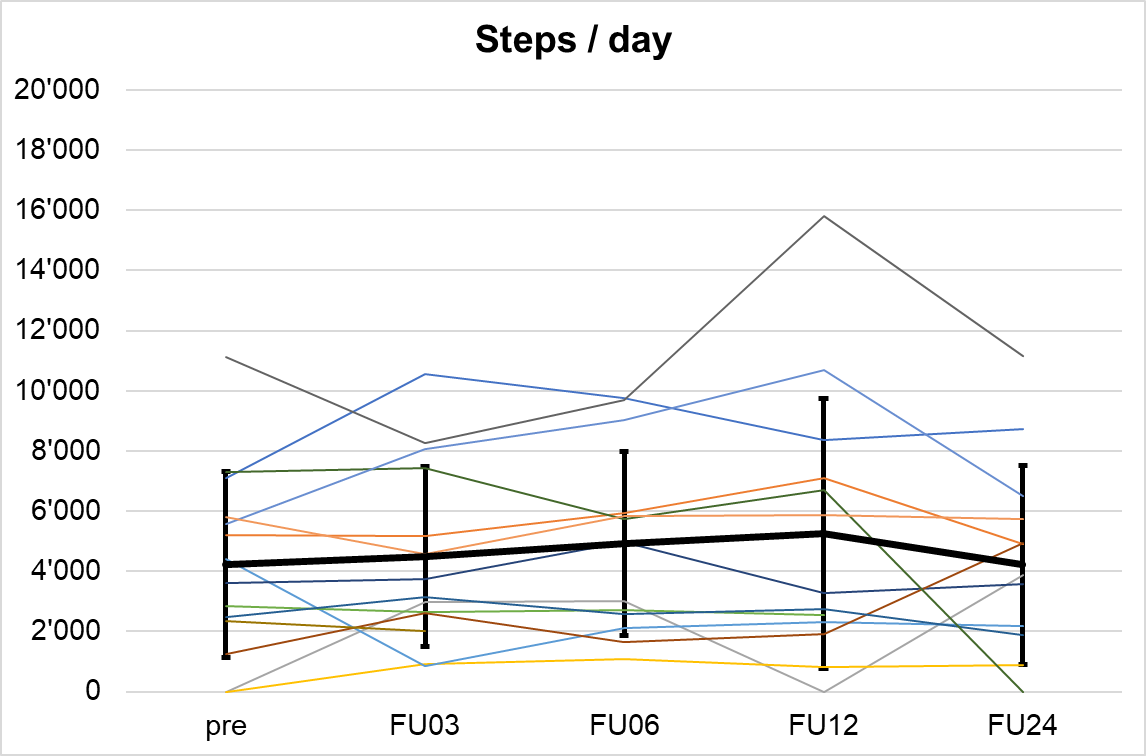 |
| 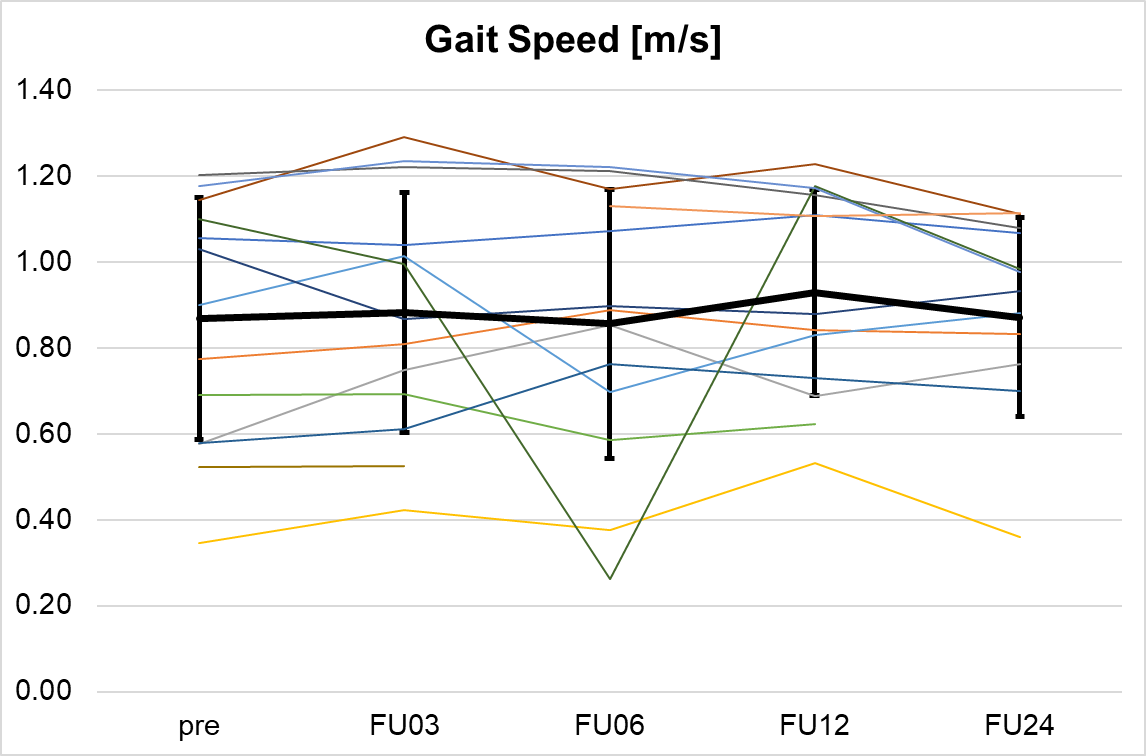 | 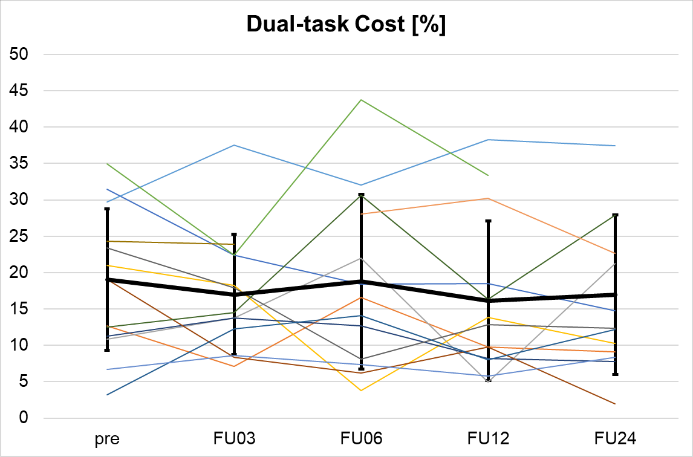 |
| 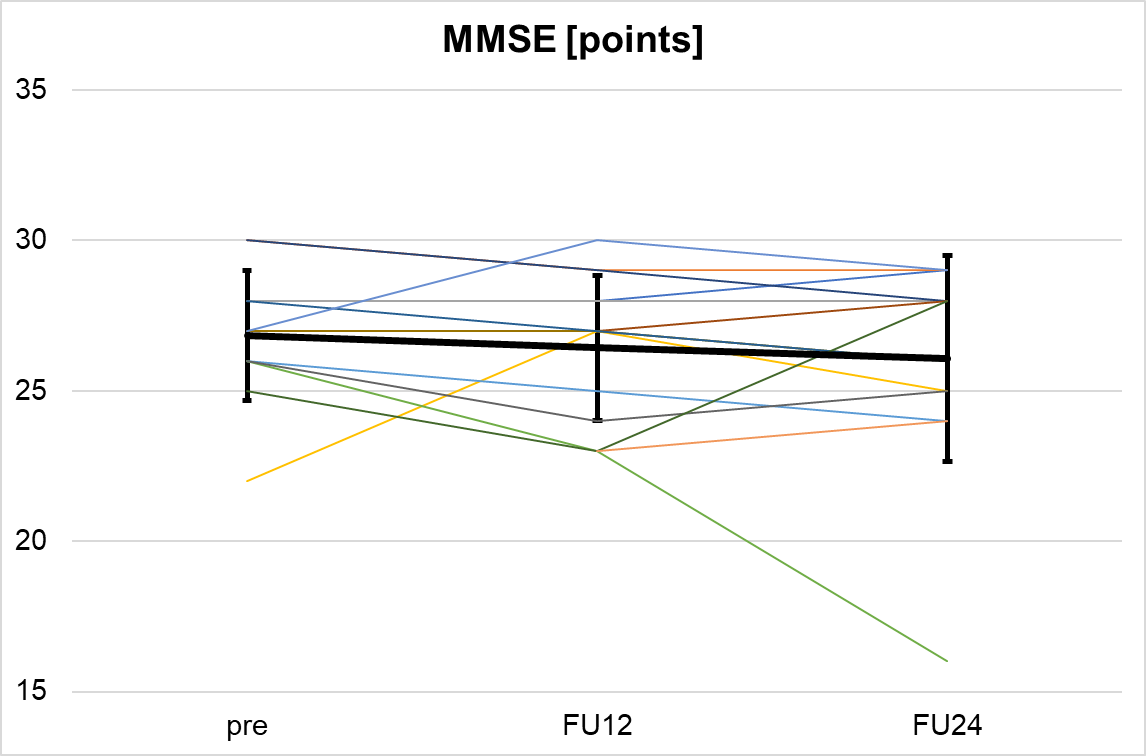 | 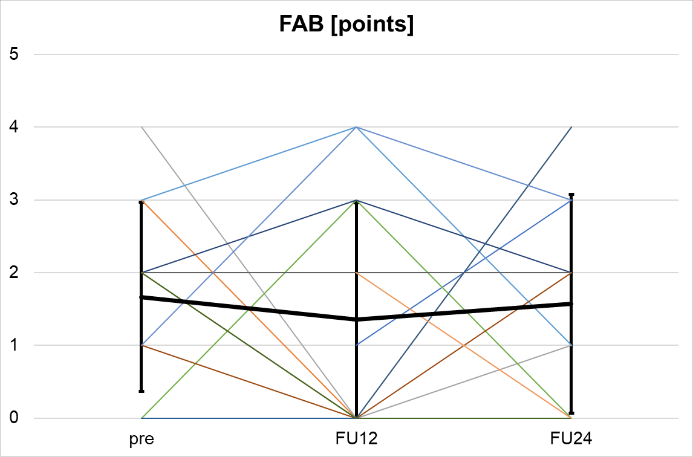 |
| 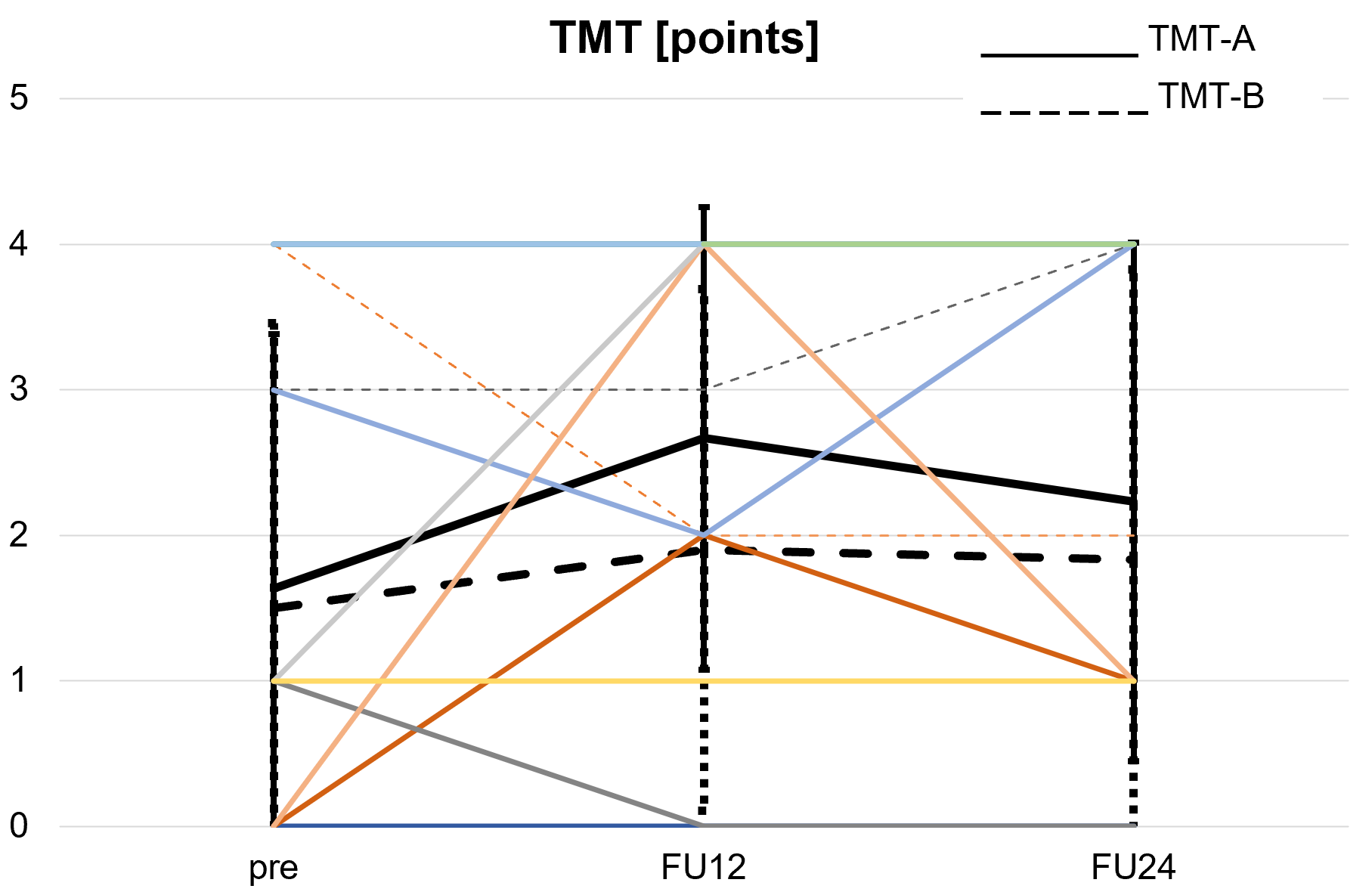 | 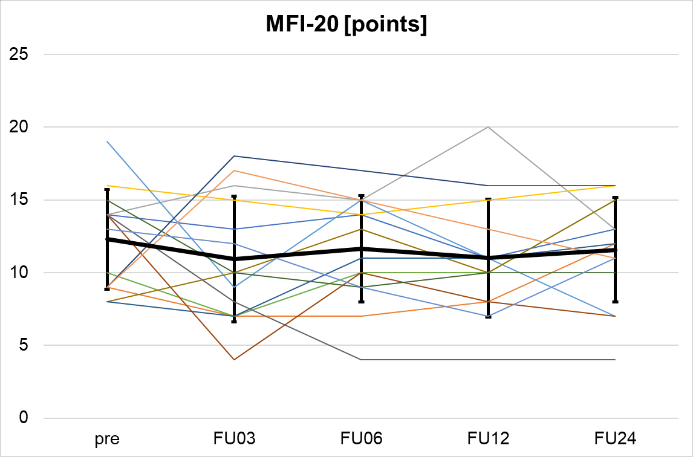 |
| 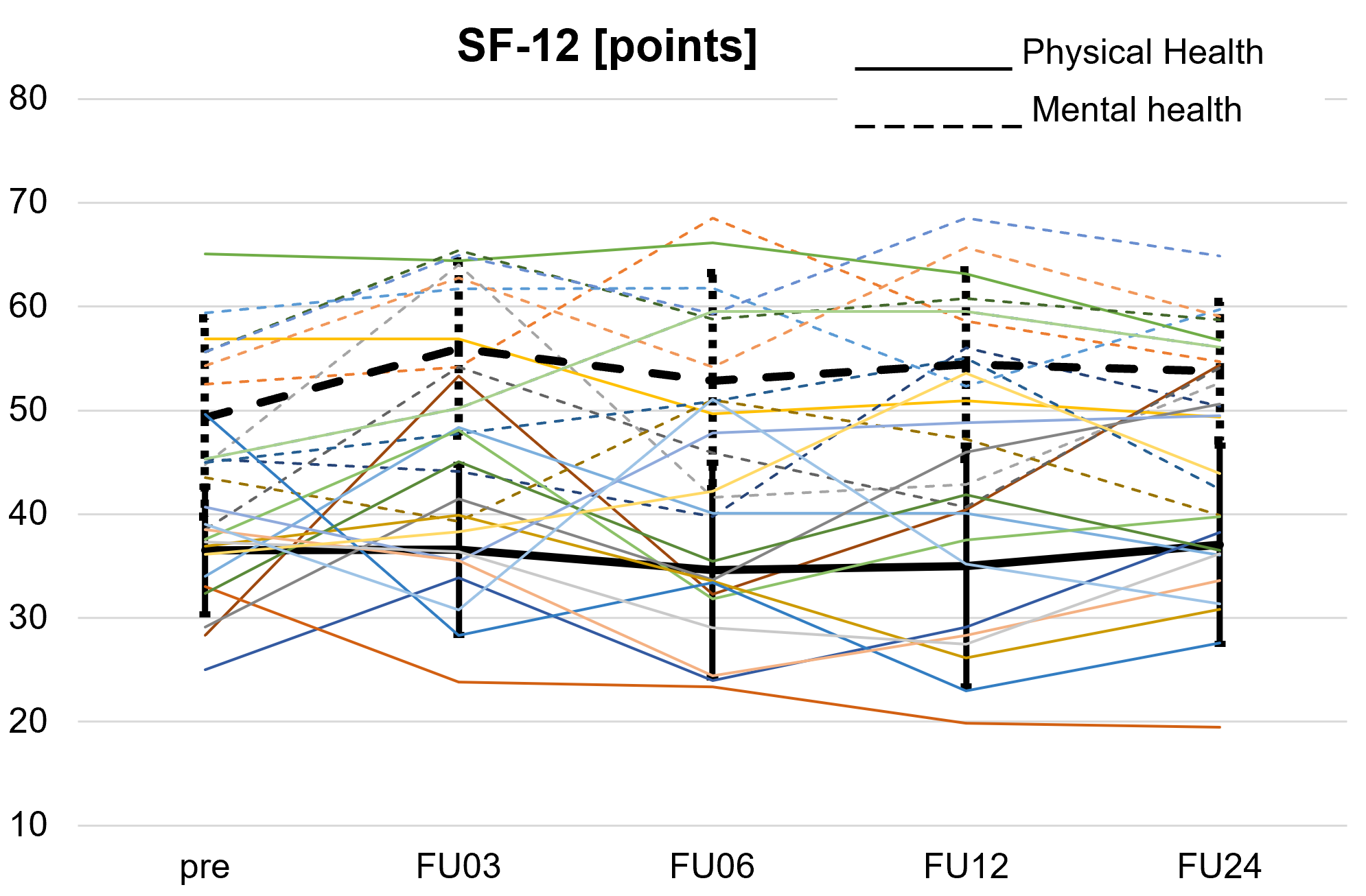 | 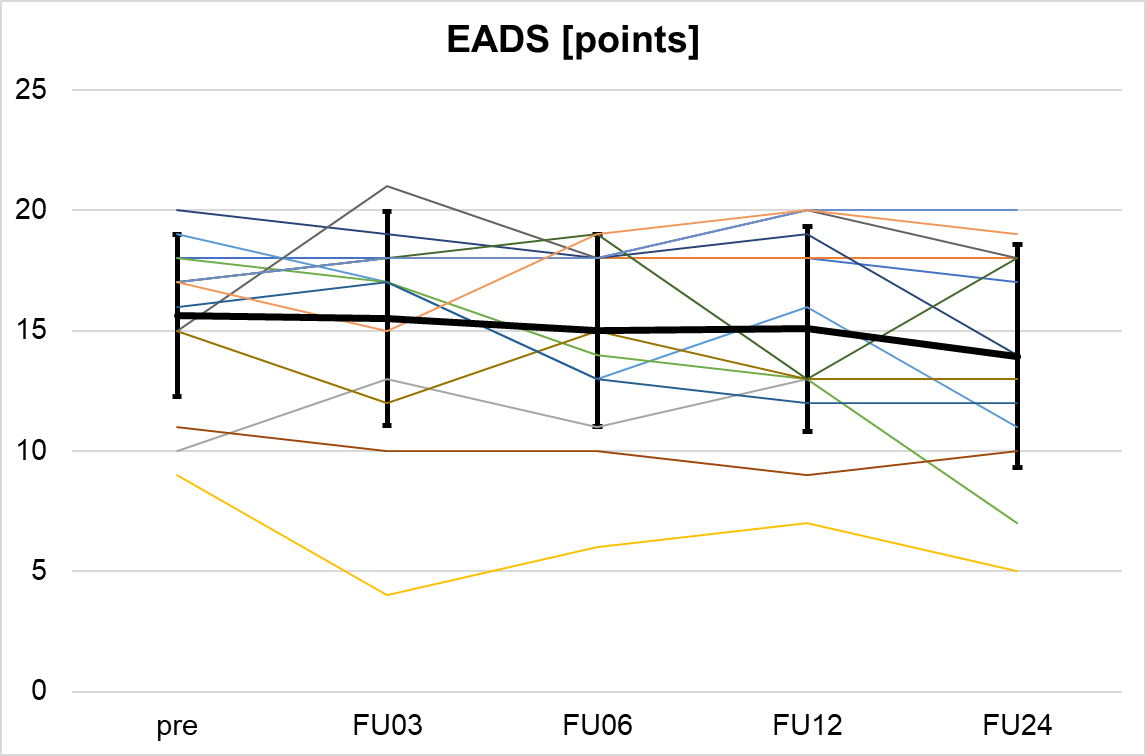 |
| 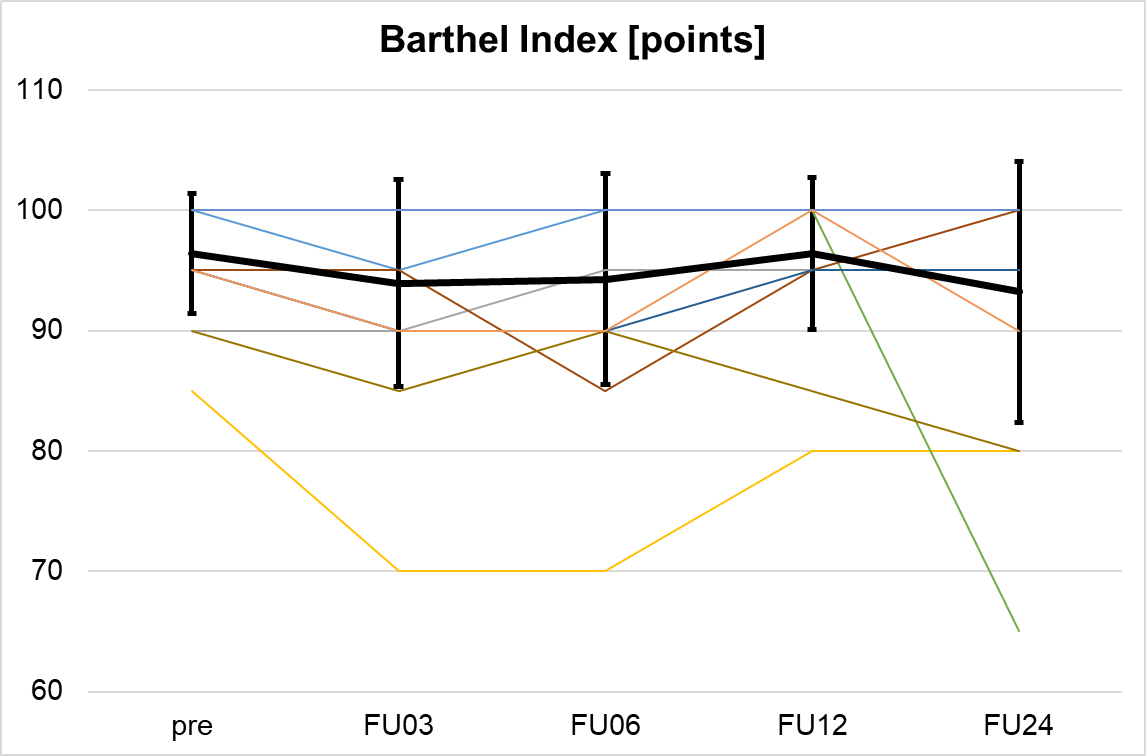 | 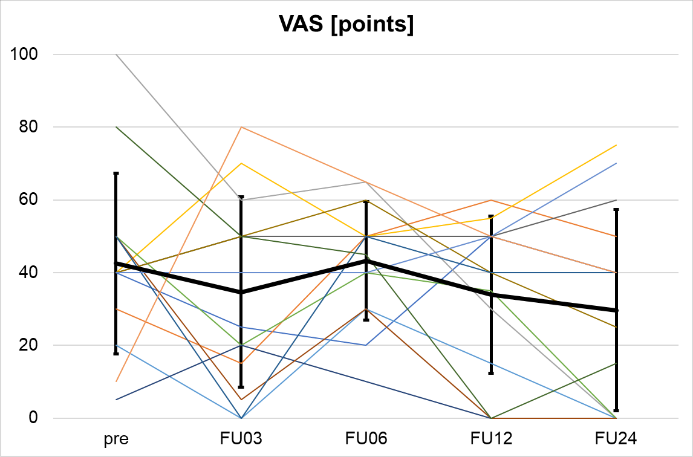 |
| 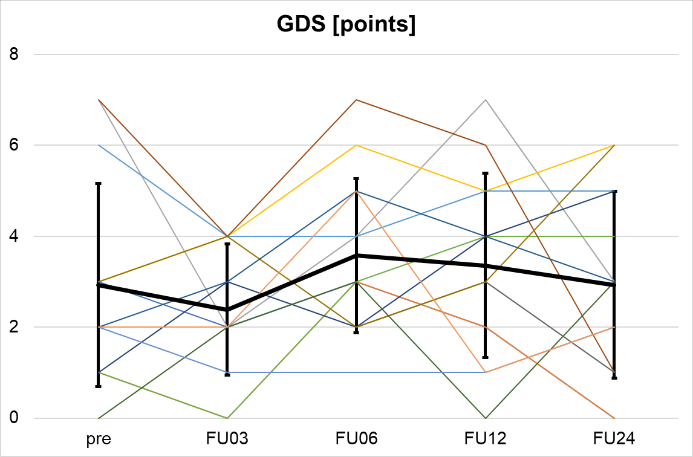 | 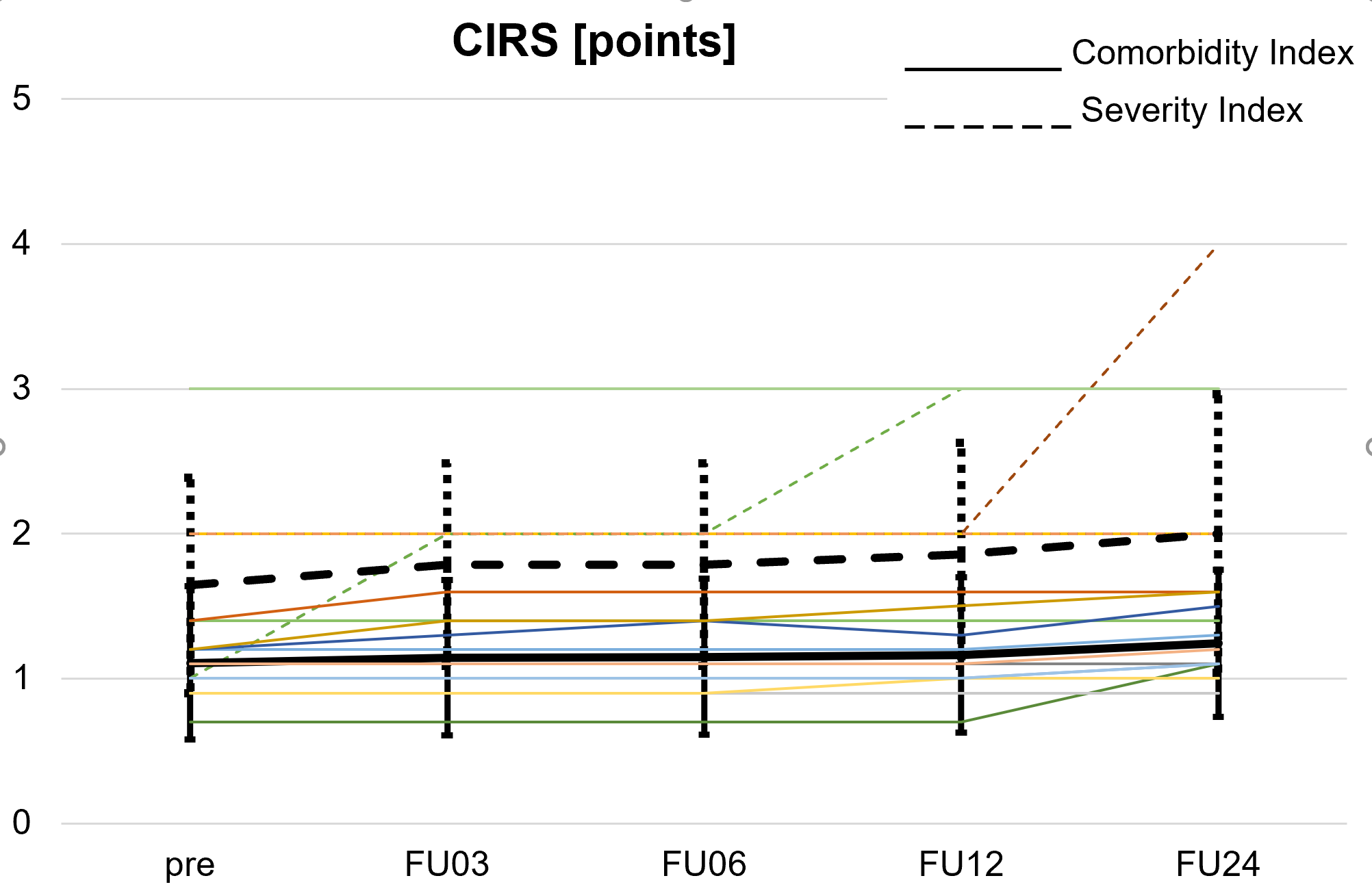 |
| 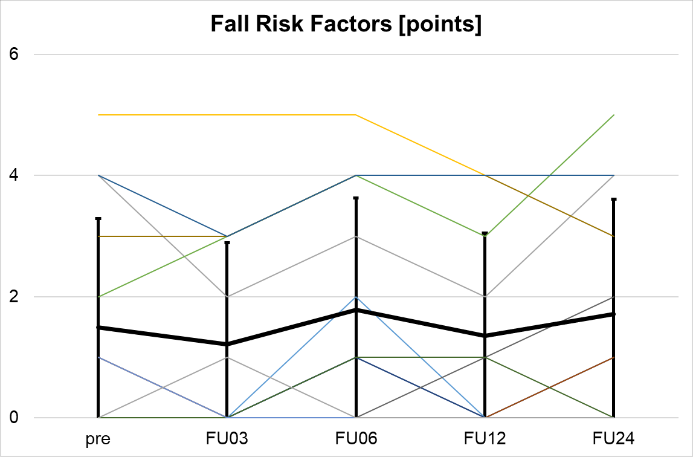 | 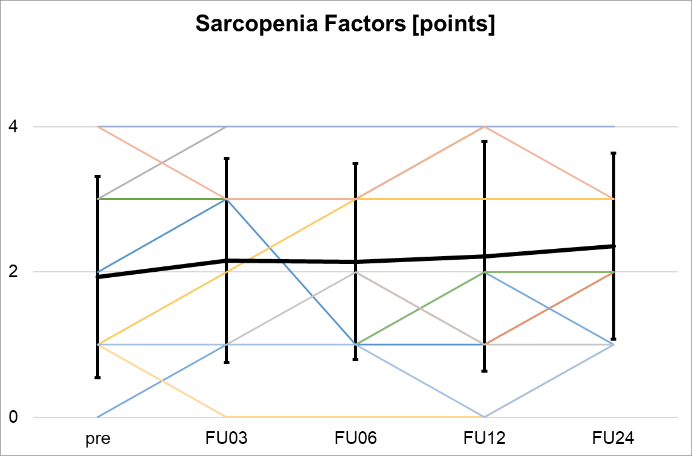 |

**S4: Evolution of physical performance tests in relation to different baseline characteristics.**

| **ETGUG < 34 s vs. ≥ 34 s**   \|  \| **Fit (n=9)** \| **Frail (n=5)** \| \| --- \| --- \| --- \| \| **POMA** \| 0.51 (0.83) \| 0.50 (0.65) \| \| **ETGUG** \| 0.44 (0.86) \| 0.47 (0.89) \| \| **SPPB** \| 0.01 (4.6) \| 0.45 (0.94) \| \| **Speed** \| 0.36 (1.02) \| 0.23 (2.52) \| | **SPPB ≥ 10 vs. < 10**   \|  \| **Fit (n=7)** \| **Frail (n=7)** \| \| --- \| --- \| --- \| \| **POMA** \| 0.29 (1.36) \| 0.45 (0.81) \| \| **ETGUG** \| 0.54 (0.58) \| 0.40 (0.93) \| \| **SPPB** \| 0.01 (6.04) \| 0.25 (1.67) \| \| **Speed** \| 0.31 (1.30) \| 0.14 (2.50) \| |
| --- | --- | --- | --- | --- | --- | --- | --- | --- | --- | --- | --- | --- | --- | --- | --- | --- | --- | --- | --- | --- | --- | --- | --- | --- | --- | --- | --- | --- | --- | --- | --- |
| **Handgrip normal vs. low**   \|  \| **Normal (n=4)** \| **Week (n=10)** \| \| --- \| --- \| --- \| \| **POMA** \| 0.54 (0.67) \| 0.43 (0.88) \| \| **ETGUG** \| 0.37 (1.16) \| 0.65 (.33) \| \| **SPPB** \| 0.33 (1.34) \| 0.04 (3.28) \| \| **Speed** \| 0.55 (0.71) \| 0.39 (0.91) \| | **Gait speed normal vs. < 0.8 m/s**   \|  \| **Normal (n=8)** \| **Low (n=6)** \| \| --- \| --- \| --- \| \| **POMA** \| 0.67 (0.56) \| 0.70 (0.25) \| \| **ETGUG** \| 0.29 (1.4) \| 0.30 (3.9) \| \| **SPPB** \| 0.04 (3.6) \| 0.69 (0.28) \| \| **Speed** \| 0.39 (1.06) \| 0.21 (8.4) \| |
| **> 1 item in fall risk or sarcopenia vs. max 1 item**   \|  \| **Fit (n=8)** \| **Frail (n=6)** \| \| --- \| --- \| --- \| \| **POMA** \| 0.55 (0.74) \| 0.60 (0.43) \| \| **ETGUG** \| 0.44 (0.85) \| 0.45 (0.78) \| \| **SPPB** \| 0.01 (4.71) \| 0.50 (0.78) \| \| **Speed** \| 0.31 (1.23) \| 0.11 (3.83) \| | **No-faller vs. faller**   \|  \| **No faller (n=6)** \| **Faller (n=8)** \| \| --- \| --- \| --- \| \| **POMA** \| 0.28 (1.43) \| 0.43 (0.91 \| \| **ETGUG** \| 0.72 (0.50) \| 0.28 (1.42) \| \| **SPPB** \| 0.10 (2.40) \| 0.10 (2.30) \| \| **Speed** \| 0.61 (0.49) \| 0.54 (0.59) \| |
| **CRP ≤ 30 mg/L vs. > 30 mg/L**   \|  \| **Normal (n=11)** \| **High (n=3)** \| \| --- \| --- \| --- \| \| **POMA** \| 0.28 (1.4) \| 0.53 (0.85) \| \| **ETGUG** \| 0.45 (0.93) \| 0.39 (1.21) \| \| **SPPB** \| 0.01 (4.42) \| 0.09 (4.24) \| \| **Steps** \| 0.63 (0.61) \| -1 part \| \| **Speed** \| 0.93 (0.16) \| 0.42 (1.04) \| | **Albumin ≥ 30 g/L vs. < 30 g/L**   \|  \| **Normal (n=11)** \| **Low (n=3)** \| \| --- \| --- \| --- \| \| **POMA** \| 0.62 (0.66) \| 0.56 (0.56) \| \| **ETGUG** \| 0.71 (0.53) \| 0.58 (0.59) \| \| **SPPB** \| 0.01 (4.3) \| 0.67 (0.32) \| \| **Steps** \| 0.31 (1.3) \| -1 part \| \| **Speed** \| 0.54 (0.57) \| 0.72 (0.23) \| |

**S5: STROBE Statement—Checklist of items that should be included in reports of cohort studies**

|  | **Item No** | **Recommendation** | **Page No** |
| --- | --- | --- | --- |
| **Title and abstract** | 1 | (*a*) Indicate the study’s design with a commonly used term in the title or the abstract | 1 |
|  |  | (*b*) Provide in the abstract an informative and balanced summary of what was done and what was found | 1-22 |
| **Introduction** | | | |
| Background/rationale | 2 | Explain the scientific background and rationale for the investigation being reported | 2 – 3 |
| Objectives | 3 | State specific objectives, including any prespecified hypotheses | 3 |
| **Methods** | | | |
| Study design | 4 | Present key elements of study design early in the paper | 3 |
| Setting | 5 | Describe the setting, locations, and relevant dates, including periods of recruitment, exposure, follow-up, and data collection | 3-4 |
| Participants | 6 | (*a*) Give the eligibility criteria, and the sources and methods of selection of participants. Describe methods of follow-up | 3-4 |
|  |  | (*b*) For matched studies, give matching criteria and number of exposed and unexposed | – |
| Variables | 7 | Clearly define all outcomes, exposures, predictors, potential confounders, and effect modifiers. Give diagnostic criteria, if applicable | 4 – 5 |
| Data sources/measurement | 8* | For each variable of interest, give sources of data and details of methods of assessment (measurement). Describe comparability of assessment methods if there is more than one group | 4 – 5 |
| Bias | 9 | Describe any efforts to address potential sources of bias | – |
| Study size | 10 | Explain how the study size was arrived at | 5 |
| Quantitative variables | 11 | Explain how quantitative variables were handled in the analyses. If applicable, describe which groupings were chosen and why | 5 – 6 |
| Statistical methods | 12 | (*a*) Describe all statistical methods, including those used to check for confounding | 5 – 6 |
|  |  | (*b*) Describe any methods used to examine subgroups and interactions | 5-6 |
|  |  | (*c*) Explain how missing data were addressed | 5-6 |
|  |  | (*d*) If applicable, explain how loss to follow-up was addressed | 5-6 |
|  |  | (*e*) Describe any sensitivity analyses | – |
| **Results** | | |  |
| Participants | 13* | (a) Report numbers of individuals at each stage of study – e.g. numbers potentially eligible, examined for eligibility, confirmed eligible, included in the study, completing follow-up, and analysed | 6 – 7 |
|  |  | (b) Give reasons for non-participation at each stage | 6 – 7 |
|  |  | (c) Consider use of a flow diagram | 6 |
| Descriptive data | 14* | (a) Give characteristics of study participants (e.g. demographic, clinical, social) and information on exposures and potential confounders | 6-7 |
|  |  | (b) Indicate number of participants with missing data for each variable of interest | 6-7 |
|  |  | (c) Summarise follow-up time (eg, average and total amount) | 6-7 |
| Outcome data | 15* | Report numbers of outcome events or summary measures over time | 6-7 |
| Main results | 16 | (*a*) Give unadjusted estimates and, if applicable, confounder-adjusted estimates and their precision (eg, 95% confidence interval). Make clear which confounders were adjusted for and why they were included | 6-7 |
|  |  | (*b*) Report category boundaries when continuous variables were categorized | 6-7 |
|  |  | (*c*) If relevant, consider translating estimates of relative risk into absolute risk for a meaningful time period | – |
| Other analyses | 17 | Report other analyses done – e.g. analyses of subgroups and interactions, and sensitivity analyses | 6-7 |
| **Discussion** |  |  |  |
| Key results | 18 | Summarise key results with reference to study objectives | 8-9 |
| Limitations | 19 | Discuss limitations of the study, taking into account sources of potential bias or imprecision. Discuss both direction and magnitude of any potential bias | 9 |
| Interpretation | 20 | Give a cautious overall interpretation of results considering objectives, limitations, multiplicity of analyses, results from similar studies, and other relevant evidence | 8-9 |
| Generalisability | 21 | Discuss the generalisability (external validity) of the study results | 8-9 |
| **Other information** |  |  |  |
| Funding | 22 | Give the source of funding and the role of the funders for the present study and, if applicable, for the original study on which the present article is based | 10 |

*Give information separately for exposed and unexposed groups.

**Note:** An Explanation and Elaboration article discusses each checklist item and gives methodological background and published examples of transparent reporting. The STROBE checklist is best used in conjunction with this article (freely available on the websites of PLoS Medicine at http://www.plosmedicine.org/, Annals of Internal Medicine at http://www.annals.org/, and Epidemiology at http://www.epidem.com/). Information on the STROBE Initiative is available at http://www.strobe-statement.org.
